# Polysaccharide from *Artocarpus heterophyllus* Lam. Pulp Ameliorates Cyclophosphamide-Induced Intestinal Damage by Regulating Gut Microbiota and Co-Metabolites

**DOI:** 10.3390/foods15010138

**Published:** 2026-01-02

**Authors:** Zhenyu He, Yunlong Li, Jun Yang, Chuan Li, Wei Wang, Yanjun Zhang, Huawei Chen, Jianjie Li, Jun Cao, Kexue Zhu

**Affiliations:** 1 School of Food Science and Engineering, Hainan University, Haikou 570228, China; 2 Spice and Beverage Research Institute, Chinese Academy of Tropical Agricultural Sciences, Wanning 571533, China; 3 School of Biological and Chemical Engineering, Zhejiang University of Science and Technology, Hangzhou 310023, China; 4 Key Laboratory of Processing Suitability and Quality Control of the Special Tropical Crops of Hainan Province, Wanning 571533, China; 5 National Center of Important Tropical Crops Engineering and Technology Research, Wanning 571533, China

**Keywords:** *Artocarpus heterophyllus* Lam., polysaccharide, gut microbiota, metabolomics, short-chain fatty acids

## Abstract

Background: Polysaccharides modulate host health by interacting with gut microbiota and reshaping the host–microbial metabolome, potentially facilitating immune regulation. Methods: This study evaluated the modulatory effect of *Artocarpus heterophyllus* Lam. (jackfruit) polysaccharide (JFP-Ps) against cyclophosphamide (Cy)-induced immunosuppression in mice, focusing on gut microbiota modulation and metabolite changes. Results: JFP-Ps effectively increased the beneficial bacteria ratio, such as *Lactobacillus* and *Lachnospiraceae*, while inhibiting some species like *Akkermansia*. Metabolomic analysis showed that JFP-Ps notably regulated gut microbe-associated metabolites, including short-chain fatty acids (SCFAs), amino acids, bile acids, indoles, and derivatives. These metabolites were involved in various metabolic pathways, including primary bile acid synthesis and biosynthesis of phenylalanine, tyrosine, and tryptophan, along with tryptophan catabolism, purine metabolic processes, and unsaturated fatty acid production. Additionally, significant correlations between microbial groups and functional metabolites were identified. Overall, JFP-Ps exerted an immuno-modulatory effect by reshaping gut microbiota and enhancing co-metabolism with the host. Conclusions: These results provided valuable insights into host–microbiota interactions and gut microbiota-targeted intervention strategies of tropical natural bioactive polysaccharides.

## 1. Introduction

The human gastrointestinal tract harbors over 100 trillion microorganisms, encompassing bacteria, fungus, viruses, archaea, and protozoa [1]. Accumulating evidence underscores the pivotal role of gut microbiota in maintaining immune homeostasis and fostering overall well-being [2]. Importantly, the gut microbiota plays a critical role in regulating immune responses. Certain microorganisms can participate in the regulation of specific subpopulations of immune cells. Conversely, a host’s immune status can alter the characteristics of the gut microbiota, such as its species composition, abundance, and distribution [3,4]. Nicholson et al. [2] described this host–microbiota metabolic cross-talk as a dynamic and reciprocal chemical signaling network, which integrates host physiological processes with the phylogenetic diversity and functionality of the gut microbiota. Metabolites serve as key mediators of the host–microbiota relationship, which encompass numerous small molecules and play a vital role in driving microbial-host cellular signaling [1,5]. Notably, metabolites synthesized by gut microbiota are critical regulators of host cellular metabolism, proliferation, and immunity [6,7,8].

The immune system exerts a critical defense against pathogens and foreign substances. Certain diseases and pharmacological interventions can compromise host defense and induce transient or persistent immune dysfunction, termed immunosuppression [9]. Cyclophosphamide (Cy) is one of the most widely employed immunosuppressive agents and is routinely used to prevent allograft rejection and manage autoimmune diseases [10]. However, high-dose Cy may disrupt DNA replication, elicit oxidative stress, suppress the proliferation of healthy cells, and attenuate both innate and adaptive immunity [11]. Furthermore, high-dose Cy has been reported to damage the gastrointestinal mucosa, increase intestinal permeability, and facilitate translocation of harmful pathogens, thereby increasing the risk of immunodeficiency and secondary infections [12]. Currently, Cy is also widely used in research on intestinal immunity models.

*Artocarpus heterophyllus* Lam. (jackfruit) is a tropical evergreen tree [13,14]. Its fruit is the largest edible fruit in the tropical and subtropical regions [15]. Jackfruit is rich in carbohydrates, minerals, polyphenols, proteins, and vitamins, earning it the title “vegetarian’s meat” [16]. Previous studies have consistently demonstrated its multiple bioactivities, including antioxidant, anti-viral, anti-tumor, anti-inflammatory, and immuno-modulatory activity [15,17,18,19]. A water-soluble polysaccharide derived from *Artocarpus heterophyllus* Lam. (jackfruit) pulp (JFP-Ps) is recognized for its notable bioactivity, biocompatibility, and non-toxicity. Our previous results showed that JFP-Ps contains rhamnose, arabinose, galactose, glucose, xylose, and galacturonic acid [19], with multiple beneficial properties, such as effective immunomodulatory, potent antioxidant, and significant anti-inflammatory activities [20,21]. Furthermore, JFP-Ps has been shown to alleviate obesity in rats by modulating gut microbiota homeostasis [22]. However, no previous study has profiled the co-regulation of metabolites and microbiota under JFP-Ps intervention in Cy-induced immunosuppression. This study seeks to assess the immune-modulating effect of JFP-Ps on the intestinal microbiome and metabolomic profiles in Cy-induced immunosuppressed mice by exploring the host–microbiota metabolic axis.

## 2. Materials and Methods

### 2.1. Materials and Reagents

JFP-Ps were extracted according to our previously reported method. Briefly, fully ripe jackfruit was harvested, the pulp was collected and cut into pieces, ground, and soaked in 80% anhydrous ethanol for 24 h. The precipitate was then collected and subjected to hot water extraction at 90 °C for 2.5 h. The aqueous extract was concentrated and four volumes of ethanol were added to precipitate the crude polysaccharides overnight. The precipitate was deproteinized using the Sevag method, followed by purification via a Sephacryl™ S-400 HR column (1.6 × 60 cm). The JFP-Ps product was obtained by freeze-drying. The FTIR spectra indicated that JFP-Ps showed several bands in the polysaccharide region of 1200–850 cm^−1^. The HPAEC-PAD chromatogram showed that JFP-Ps were composed of rhamnose, arabinose, galactose, glucose, xylose, and galacturonic acid, with purity above 89.58% and an average molecular weight of 1668 kDa [19].

Chromatographic-grade methanol and acetonitrile were purchased from Merck (Darmstadt, Germany). Methoxylamine hydrochloride and formic acid were purchased from Sigma-Aldrich (St. Louis, MO, USA). N-methyl-N-(trimethylsilyl) trifluoroacetamide (MSTFA) was obtained from Aladdin (Shanghai, China). Pyridine was obtained from Xiya Reagents (Chengdu, China). Cy was obtained from Baxter Oncology GmbH (Halle, Germany).

### 2.2. Animals and Experimental Design

Thirty specific pathogen-free (SPF) male mice of the BALB/c strain, aged 5 weeks and with a body weight (BW) of 18 ± 2 g, were supplied by Hunan Sleek Jingda Laboratory Animal Co. Ltd. (Changsha, China, Certificate No. SCXK (Xiang) 2019-0004. After acclimatization for one week in a controlled environment (25 ± 1 °C, 55 ± 15% humidity, 12/12 h light/dark cycle), the animals were randomly assigned to five groups (*n* = 6) using a lottery method: the normal control (NC) group received sterile saline; the model control (MC) group was intraperitoneally administered Cy at 100 mg/kg body weight for three consecutive days; and the JFP-Ps treatment groups (JFP-Ps-L, JFP-Ps-M, JFP-Ps-H) were given Cy as above, followed by daily oral gavage of JFP-Ps at 50, 100, and 200 mg/kg body weight for seven days, respectively. The mice were euthanized 24 h after the last gavage. The fresh fecal, organ, and tissue samples were stored by freezing them at −80 °C. Sample size was based on published studies using Cy-induced dysbiosis models reporting significant SCFA and microbiota composition changes, with *n* = 6 [23]. Our study should therefore be considered exploratory regarding small effect sizes. The mice were housed individually in cages at a room temperature of 23 °C, with free access to food and water. The feed composition consisted of 18.0% crude protein, 4.0% crude fat, 5.0% crude fiber, 10.0% moisture, 8.0% ash, 1.8% calcium, and 1.0% phosphorus. License number: SCXK (Xiang) 2020-0006. The animal experiment strictly adhered to the National Guide for the Care and Use of Laboratory Animals and received approval (HNUAUCC-2021-00118) from the Animal Ethics Committee of Hainan University, China.

### 2.3. Determination of SCFAs in the Feces

According to the approach that we had previously taken [24], the concentration of SCFAs in feces was measured by gas chromatography (GC). Fecal samples (100 mg) were dissolved in 500 µL of ultrapure water and vortex-mixed. To remove particulate matter, the sample was first centrifuged (12,000× *g*, 20 min). The clarified supernatant was then collected and sterilized by filtration through a 0.22 μm pore-size membrane. SCFA concentrations (mg/g) were then quantified using an Agilent 7890A (Agilent Technologies, Santa Clara, CA, USA) gas chromatography system. The chromatography column used was an HP-FFAP (30 m × 0.32 mm × 0.25 μm), with the injector temperature set at 240 °C. The carrier gas was nitrogen at a flow rate of 19 mL/min, while the air and hydrogen flow rates were 350 mL/min and 30 mL/min, respectively.

### 2.4. 16S rDNA Microbial Community Analysis

Genomic DNA was isolated from feces utilizing the DNeasy PowerSoil kit (QIAGEN, Hilden, Germany) in accordance with the specifications. Targeting the bacterial 16S rRNA V3–V4 hypervariable region, polymerase chain reaction (PCR) amplification was performed with the primers and cycling conditions described by Zeng et al. [22]. The reaction mixture consisted of 10 μL of 2× Pro Taq enzyme, 0.8 μL each of forward and reverse primers (5 μM), template DNA (10 ng/μL), and nuclease-free water added to a final volume of 20 μL. The thermal cycling protocol for RT-qPCR included an initial denaturation at 95 °C for 3 min, followed by 29 cycles of denaturation at 95 °C for 30 s, annealing at 55 °C for 30 s, and extension at 72 °C for 45 s, with a final extension at 72 °C for 10 min and storage at 10 °C. PCR amplification products were detected on a 2% agarose gel using a DYY-6C electrophoresis analyzer. Purification of PCR products was performed with the AxyPrep DNA Gel Recovery Kit (AXYGEN, Union City, CA, USA) in accordance with the supplier’s guidelines, and their concentration was determined using the QuantiFluor™-ST Blue Fluorescence Quantitative System (Promega, Madison, WI, USA).

DNA sequencing libraries were prepared with the TruSeqTM DNA Sample Prep Kit (Illumina, San Diego, CA, USA). Purified amplicons were subsequently subjected to paired-end sequencing on an Illumina MiSeq platform (Illumina, San Diego, CA, USA). The data were filtered to retain only those OTUs that were present in at least three samples and had a total read count of no less than 5. α-diversity analysis at the OTU level was conducted to assess species richness and diversity within microbial communities. Specifically, the Chao1 and ACE indices primarily reflect species richness, while the Simpson and Shannon indices primarily characterize species diversity. Subsequent bioinformatic analysis was conducted using Majorbio “www.majorbio.com (accessed on 20 June 2023)”.

### 2.5. Metabolomics Analysis

Fecal samples from each group (50 mg) were combined with 600 μL of the extraction solution (methanol: acetonitrile: water = 2:2:1, pre-chilled at 4 °C), homogenized for 1 min at 60 Hz using a freezer mill, vortexed for 30 s, and sonicated at room temperature for 10 min. The samples were centrifuged at 14,000× g, 4 °C for 15 min, and the supernatants were collected and filtered through a 0.22 μm organic filter membrane. Data was acquired using a UPLC-Q/TOF-MS/MS system.

Chromatographic separation was achieved using an Agilent ZORBAX RRHT Eclipse Plus C18 column (3.0 × 150 mm, 1.8 μm) held at 35 °C, with a mobile phase of (A) water with 0.1% formic acid and (B) acetonitrile, delivered at 0.4 mL/min. Using an injection volume of 3 μL, the following gradient program was applied: 0–2 min, 2% B; 2–8 min, 2–10% B; 8–23 min, 10–14% B; 23–40 min, 14–70% B; 40–40.5 min, 70–100% B; 40.5–42 min, 100% B. The total elution time was 42 min. Mass spectrometry detection was conducted utilizing an Agilent 6530 quadrupole time-of-flight mass spectrometer for both positive and negative ion scanning; ionization mode was ESI. The mass spectrometer scan range was set to 100–1000 *m*/*z*. The fragmentation voltage applied was 140 V, while the drying gas temperature was maintained at 250 °C. Additionally, the following MS parameters were applied: drying gas flow, 8.0 L/min; nebulizer pressure, 40 psig; and sheath gas temperature, 325 °C.

The data were initially analyzed using Mass Hunter qualitative analysis software (version B.06.01, Agilent Technologies, Santa Clara, CA, USA), converted to a common data format, and the metabolites were identified using Mass Profiler Professional software (version B.14.0, Agilent Technologies, Santa Clara, CA, USA). The gathered data were loaded into SIMCA-P software (version 14.1, Germany) for subsequent multivariate statistical analysis. The significance value of the predictor variables (VIP > 1), the difference multiplier (|FC| > 2), and the *p*-value (*p* < 0.05) of one-way ANOVA of the OPLS-DA model were used as screening criteria for differential small-molecule compounds. Cluster analysis and pathway analysis of potential markers were performed on MetOrigin 5.0 “http://metorigin.met-bioinformatics.cn/ (accessed on 25 June 2023)”.

### 2.6. Statistical Analysis

Statistical analyses were performed with SPSS 22.0, applying either Student’s t-test or one-way ANOVA followed by LSD post hoc tests, as appropriate for the data structure. The data were expressed as the mean ± standard error of the mean (SEM). A *p*-value of less than 0.05 was deemed statistically significant. The biomarkers and microbial diversity data obtained from the metabolomics screening were integrated by the webserver MetOrigin “http://metorigin.met-bioinformatics.cn/ (accessed on 25 June 2023). The Sankey network was employed to visualize the correlation between the obtained biomarkers and microorganisms.

## 3. Results

### 3.1. JFP-Ps Regulated the Intestinal Microbiota Structure of Immunosuppressed Mice

#### 3.1.1. Species Diversity of the Gut Microbiota

The gut microbiome serves as a key modulator of immune function. Fecal microbiota diversity and composition were examined using 16S rDNA high-throughput sequencing. A total of 1,514,187 high-quality 16S rDNA sequences were acquired from murine excrement, with an average sequence length of approximately 1502 bp. Based on 97% sequence similarity, the obtained sequences were clustered into 1019 operational taxonomic units (OTUs). The Sobs rarefaction curve was clear (Figure S1A), and the Shannon curve was smooth and stable (Figure S1B), indicating that the sequencing of the various groups captured the bacterial communities comprehensively, and the data can accurately represent the microbial structure of the samples.

**Figure 1:**
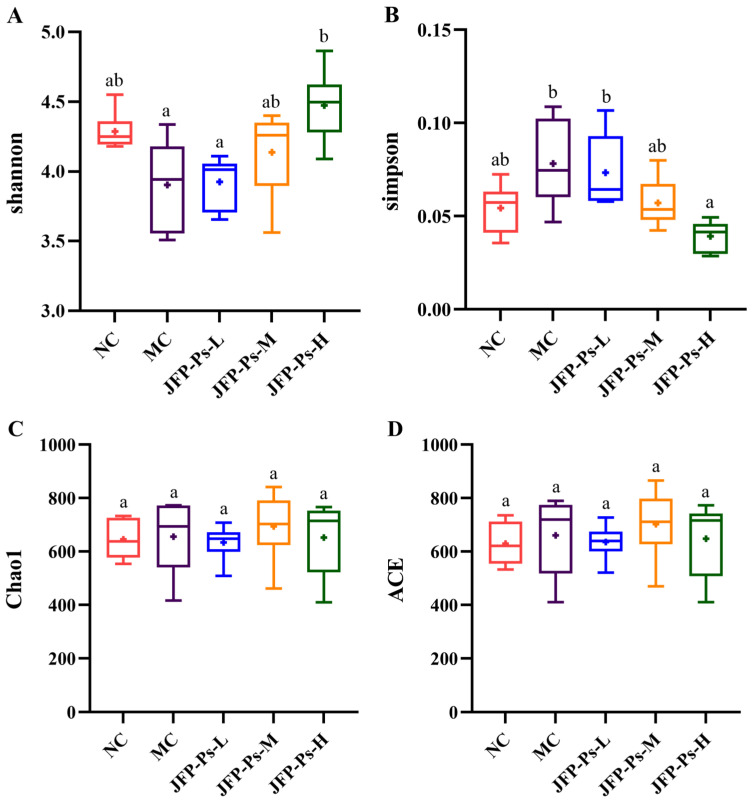
Effects of JFP-Ps on α-diversity of gut microbiota in Cy-induced immunosuppressed mice. (**A**) Shannon index, (**B**) Simpson index, (**C**) Chao1 index, (**D**) ACE index. The results of α-diversity showed that high-dose JFP-Ps treatment significantly increased microbial community diversity with good microbial community richness. Significant differences were denoted by distinct letters based on Duncan’s multiple range test at a significance level of *p* < 0.05.

α-Diversity analysis at the OTU level was performed to assess microbial community richness and diversity. The species richness of the gut microbiota was mainly reflected by the Chao1 and ACE indices, while microbial diversity was primarily reflected by the Simpson and Shannon indices. As presented in Figure 1, the α-diversity indices (Shannon and Simpson) remained comparable across the NC, MC, JFP-Ps-L, and JFP-Ps-M groups, indicating no statistically significant differences in microbial community diversity. In comparison to the MC group, the Shannon index of the JFP-Ps-H group exhibited a considerable rise (*p* < 0.05); however, the Simpson index demonstrated a significant drop (*p* < 0.05), indicating that high-dose JFP-Ps treatment significantly increased microbial community diversity. Chao and ACE indices reflected microbial community richness; Figure 1C,D show no differences among the groups, indicating good microbial community richness in all samples.

**Figure 2:**
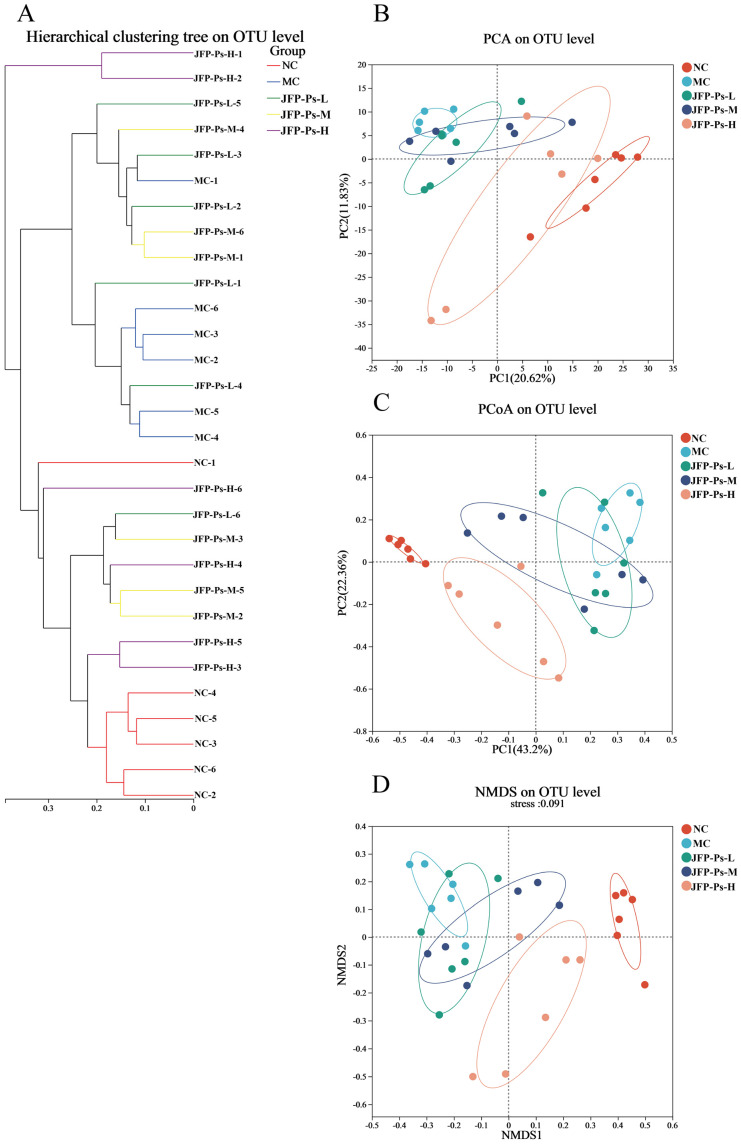
Effects of JFP-Ps on β-diversity of gut microbiota in Cy-induced immunosuppressed mice. (**A**) Cluster tree of gut microbiota at OTU level, (**B**) PCA analysis of gut microbiota at OTU level, (**C**) PCoA analysis of gut microbiota at OTU level, (**D**) NMDS analysis of gut microbiota at OTU level. The results of β-diversity showed that JFP-Ps affected the microbial community of Cy-induced immunosuppressed mice in a dose-dependent manner.

To further evaluate the effects of Cy and JFP-Ps on the microbial community, β-diversity of the microbial community was analyzed. The principal coordinates analysis (PCA) revealed differences in the microbial communities between the NC, MC, JFP-Ps-L, JFP-Ps-M, and JFP-Ps-H groups (Figure 2B). The NC and MC groups were noticeably separated, indicating considerable changes in the fecal microbiota structure of mice treated with Cy. The JFP-Ps groups were differentiated from the MC group and found to be closer to the NC group. The results suggested that JFP-Ps affected the fecal microbiota structure of immunosuppressed mice in a dose-dependent manner. Additionally, similar results were observed in sample hierarchical clustering analysis (Figure 2A), PCoA analysis (Figure 2C), and NMDS analysis (Figure 2D).

#### 3.1.2. Changes in the Fecal-Microbiota Structure

**Figure 3:**
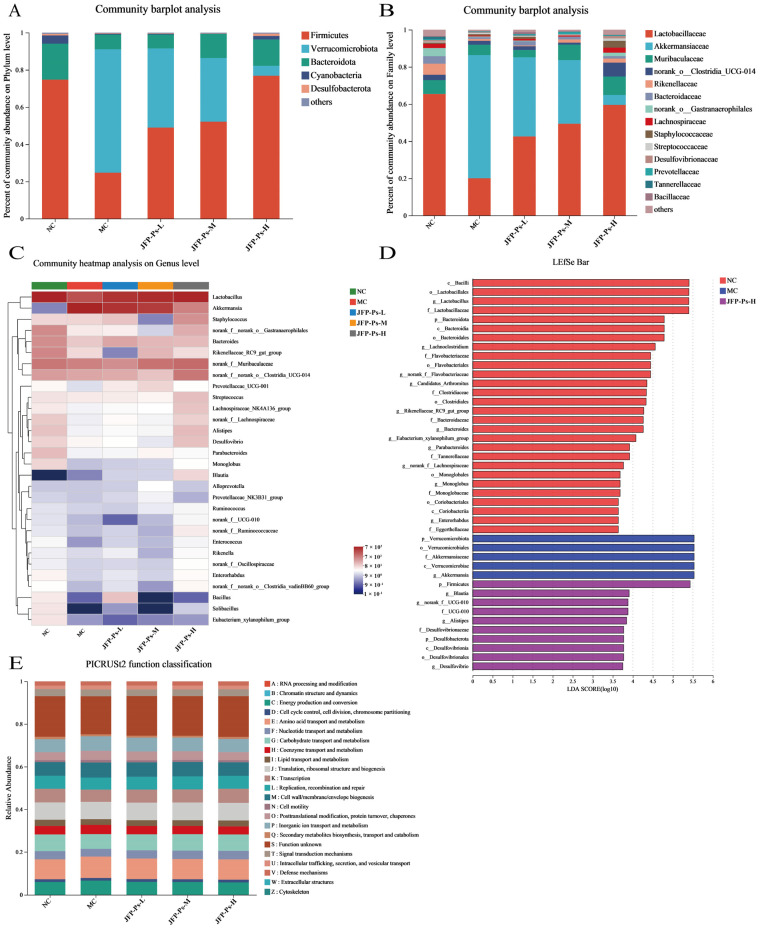
Effects of JFP-Ps on the composition of gut microbiota in Cy-induced immunosuppressed mice. Microbial community composition at the phylum level (**A**) and family level (**B**);heatmap showing the relative abundance of key genera (**C**); histogram of LDA effect values for biomarker species, LDA > 2 (**D**); prediction of PICRUSt2 function in the microbiome (**E**). The key species types of microbiota in the feces of Cy-induced immunosuppressed mice were altered by JFP-Ps.

The microbial community abundance variations from a taxonomic perspective (phylum, family, genus) were analyzed to examine the impact of JFP-Ps on the gut microbiota in Cy-induced immunosuppressed mice. At the phylum level (Figure 3A), the microbial community was dominated by the phyla *Firmicutes*, *Verrucomicrobiota*, *Bacteroidota* and *Cyanobacteria*, accounting for more than 97% of the total community. Compared with the NC group, a pronounced enrichment of *Verrucomicrobiota*, whereas the abundances of *Firmicutes* and *Bacteroidota* were concurrently reduced in Cy-induced immunosuppression mice. Interestingly, compared with the MC group, JFP-Ps significantly affected the microbial community changes caused by Cy. In particular, the JFP-Ps-H treatment almost entirely reversed the variations.

Based on the mean relative abundance, the ten predominant families were identified and presented in Figure 3B. Compared to the NC group, the intestinal microbiota of Cy treatment mice showed a decrease in the relative abundance of *Lactobacillaceae*, *Muribaculaceae*, *Bacteroidaceae*, *Rikenellaceae*, *Lachnospiraceae*, and *norank_o__Gastranaerophilales*, and an increase in the relative abundance of *Akkermansiaceae*, *norank_o__Clostridia_UCG-014*, and *Staphylococcaceae*. However, JFP-Ps treatment reversed the decrease in the relative abundance of *Lactobacillaceae*, *Muribaculaceae*, *Bacteroidaceae*, *Rikenellaceae*, *Lachnospiraceae*, and *norank_o__Gastranaerophilales*. In addition, *Akkermansiaceae* abundance was significantly depleted by JFP-Ps intervention.

Heatmap analysis was conducted at the genus level (Figure 3C). Compared with the NC group, the MC group displayed decreased relative abundance in *Lactobacillus*, *Rikenellaceae_RC9_gut_group*, *Alistipes*, *Desulfovibrio*, *norank_f__Lachnospiraceae*, *Parabacteroides*, *norank_f__ UCG-010*, *norank_f__Flavobacteriaceae*, *Candidatus_Arthromitus*, and *Lachnoclostridium*, and increased relative abundance in *Akkermansia*. *Akkermansia* exhibited the highest proportion among the MC group. Interestingly, the intervention of JFP-Ps resulted in the reversal of the aforementioned changes in the microbial community. Additionally, JFP-Ps increased the relative abundance of *Blautia*. Hence, these results suggest that JFP-Ps modulates the gut microbiota of immunosuppressed mice.

#### 3.1.3. Identification of Phenotypic Biomarkers

LEfSe analysis was conducted to determine the phenotypic biomarkers of gut microbiota in the different groups (Figure 3D). A total of 20 dominant microorganisms in the NC group were identified by LDA analysis (LDA > 2, *p* < 0.05). The top five dominant microorganism groups based on the LDA scores were *Firmicutes*, *Lactobacillales*, *Bacilli*, *Lactobacillus*, and *Lactobacillaceae*. Five dominant bacterial groups were identified in the MC group (LDA > 2, *p* < 0.05), namely *Akkermansiaceae*, *Akkermansia*, *Verrucomicrobiota*, *Verrucomicrobiales*, and *Verrucomicrobiae*. No dominant microorganisms were observed in the JFP-Ps-L and JFP-Ps-M groups. However, 10 dominant microorganisms (LDA > 2, *p* < 0.05), including *Blautia*, *Alistipes*, and *Desulfovibrionaceae* as primary microorganisms, were found in the JFP-Ps-H group. These results suggest that the intervention of JFP-Ps altered the key species types of microbiota in the feces of cy-induced immunosuppressed mice and promoted the multiplication of specific microbiota.

### 3.2. JFP-Ps Increased the Production of SCFAs

SCFAs serve as critical fuels for colonic epithelial cells and are key regulators of their proliferation and differentiation. They also significantly influence the function of subpopulations of intestinal endocrine cells, thereby impacting intestinal barrier function and host metabolism. This study examined the correlation between the efficacy of JFP-Ps in modulating the intestinal tract of immunosuppressed mice and the production of SCFAs in their feces. The results showed that the fecal SCFA content in mice treated with Cy was significantly lower than that in the NC group (Table 1, *p* < 0.05). However, after JFP-Ps treatment, the fecal SCFA content in the mice was significantly increased (Table 1, *p* < 0.05). Thus, it was concluded that the health-beneficial effect of JFP-Ps on the immunosuppressed mice’s intestinal tract was linked to the promotion of SCFA formation.

### 3.3. JFP-Ps Altered the Fecal Metabolic Profile of Immunosuppressed Mice

**Figure 4:**
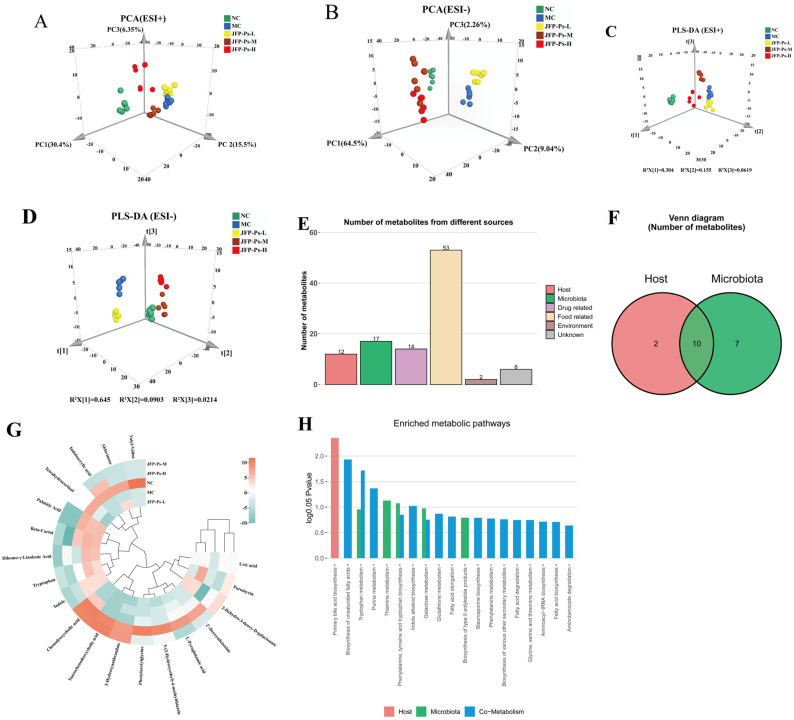
Effects of JFP-Ps on the fecal metabolic profile in Cy-induced immunosuppressive mice. PCA plots of metabolites among NC, MC, JFP-Ps-L, JFP-Ps-M, and JFP-Ps-H groups in the (**A**) positive and (**B**) negative ion models. PLD-DA plots of metabolites among NC, MC, JFP-Ps-L, JFP-Ps-M, and JFP-Ps-H groups in the (**C**) positive and (**D**) negative ion models. Bar graphs (**E**) and Venn diagrams (**F**) of the number of metabolites in different classes based on MetOrigin analysis and MPEA analysis. Biomarker heatmap (**G**) and bar plot (**H**) of the number of enriched metabolic pathways from origin-based MPEA analysis.

Intestinal microbiota metabolites are key mediators of the interaction between the microbiota and the host. As such, UPLC-Q-TOF-MS technology was employed to examine the fecal metabolome of the mice. The results showed that there was distinct separation among the different groups, indicating significant metabolic differences among them (Figure 4A–D). OPLS-DA demonstrated distinct separation between the groups and effective clustering within each sample group. The interpretation ability parameters (R^2^Y) for each group were close to 1, and the prediction ability parameters (Q^2^) were all above 0.7 (Figure S3), confirming the reliability of the model’s interpretation and prediction capabilities. Additionally, 200 permutation tests were conducted in the corresponding OPLS-DA models, and the results showed that the model did not overfit (Figure S4).

A total of 565 and 1071 metabolic features were detected in the positive and negative ionization modes, respectively (Figure S2). The volcano plot displayed variables with significant differences in the content of the different groups (Figure S5). Relative to the NC group, the MC group exhibited significant alterations in 331 metabolites. Among these, 47 were upregulated and 125 downregulated in positive ion mode, while 76 were upregulated and 83 downregulated in negative ion mode. After JFP-Ps-H intervention, 57 and 307 metabolites were upregulated, while 79 and 416 metabolites were downregulated in the positive and negative ion modes, respectively. By combining FC values > 2, VIP values > 1, and *p*-values < 0.05, 61 potential biomarkers were selected (Table S1), including peptides, amino acids, bile acids, indoles and their derivatives, and SCFAs.

To further understand the relationship between metabolite changes and gut microbiota, origin-tracing analysis was conducted on these metabolites using the MetOrigin platform’s database. As shown in Figure 4E,F, the metabolites were classified into six categories based on their sources: two host-specific metabolites, seven microbe-specific metabolites, and ten host-microbe co-metabolites. Additionally, the compounds associated with drugs (14 types), foods (53 types), the environment (2 types), and unknown sources (6 types) were identified. We concentrated on the chemicals associated with the host, microbe, and co-metabolism. The heatmap (Figure 4G) showed that Cy treatment significantly increased the levels of tryptophan, puromycin, indole, taurallocholic acid, uric acid, 2-dehydro-3-deoxy-D-galactonate, β-cortol, and dihomo-γ-linolenic acid (*p* < 0.05), while the levels of aklavinone, indoleacrylic acid, L-pyroglutamic acid, 2′-deoxyadenosine, palmitic acid, tetrahydrocortisol, and 3-hydroxyanthranilate were significantly decreased. Interestingly, the intervention of JFP-Ps partially reversed the changes in these metabolites. In addition, JFP-Ps significantly increased the levels of taurochenodeoxycholic acid and 3a,7a-dihydroxycoprostanic acid, indicating that JFP-Ps may improve immune function by intervening in the metabolism of gut microbiota and the host.

### 3.4. JFP-Ps Modulated the Metabolic Pathway of the Intestinal Microbiota

Subsequently, key biomarkers and their related metabolic pathways were elucidated by enrichment analysis. As shown in Figure 4H, 19 metabolic pathways were influenced by JFP-Ps. Among them, one host-specific pathway (primary bile acid biosynthesis) was significantly enriched. Two microbial metabolic pathways, thiamine metabolism and the production of phenylalanine, tyrosine, and tryptophan, exhibited substantial enrichment. Furthermore, four host-microbe co-metabolic pathways (biosynthesis of unsaturated fatty acids, tryptophan metabolism, purine metabolism, and indole alkaloid biosynthesis) were also significantly enriched.

**Figure 5:**
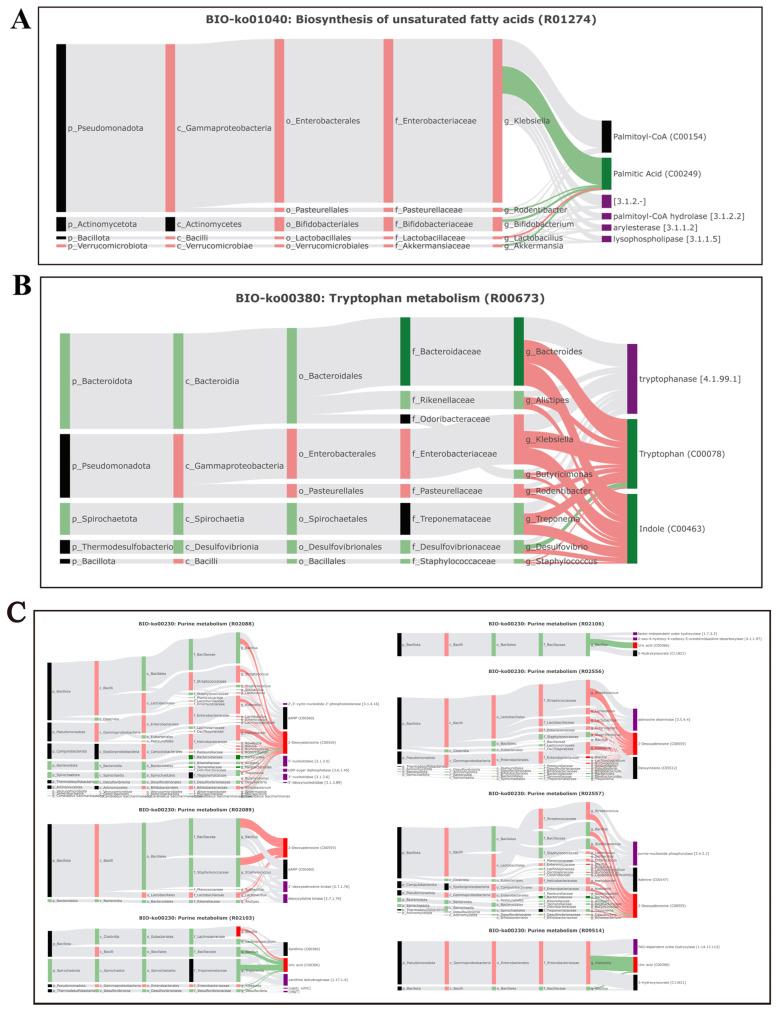

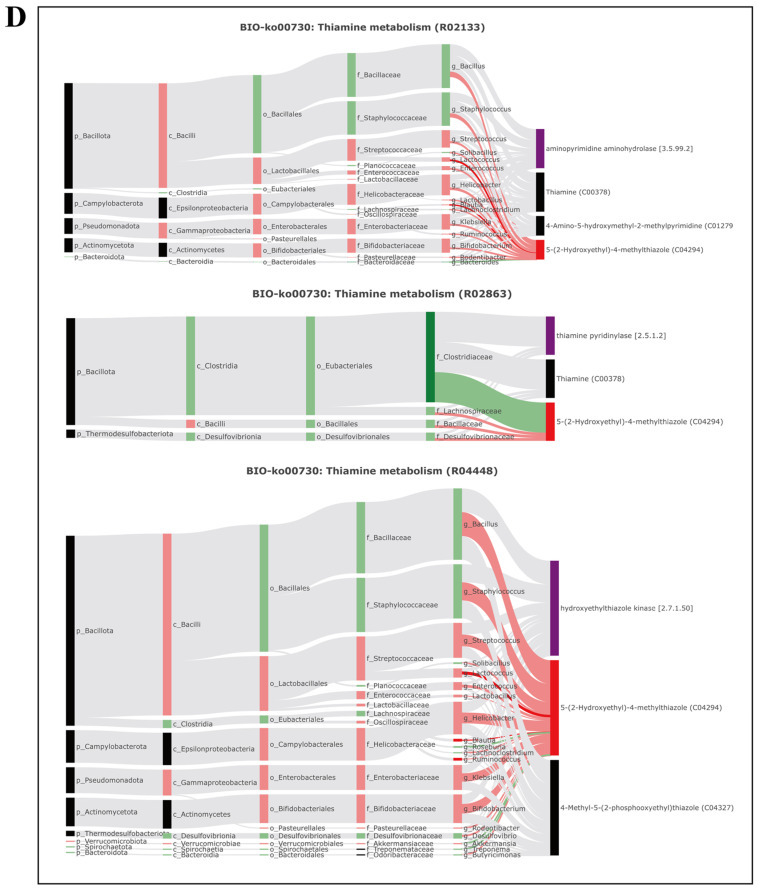

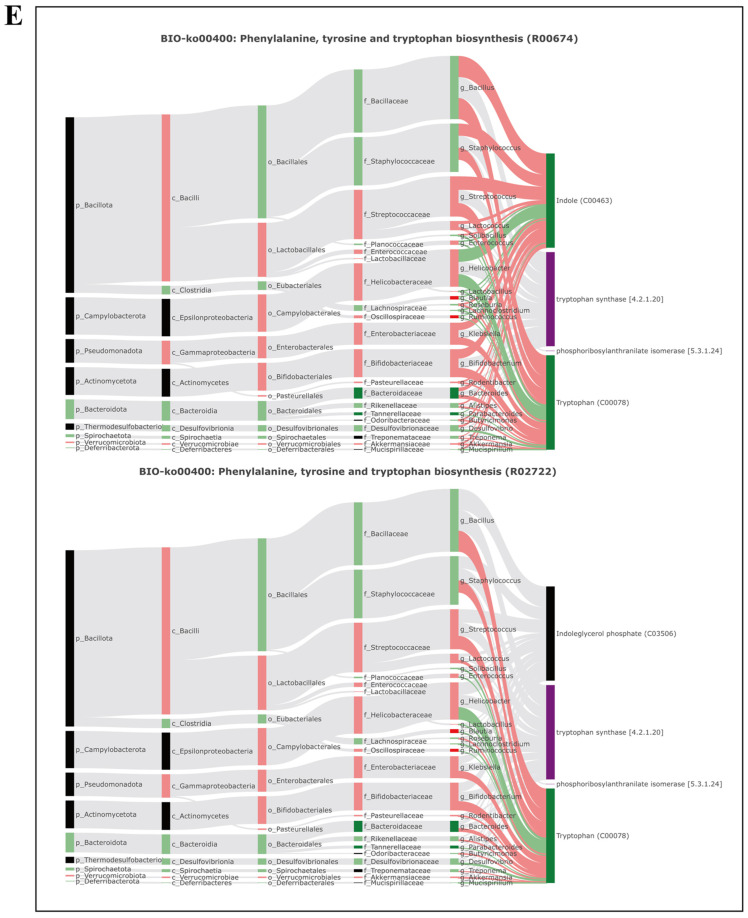
Microbiota-specific metabolic pathways, BIO-Sankey network diagrams, and network summary analysis revealed close associations between microbiota and metabolites. (**A**) Biosynthesis of unsaturated fatty acids (BIO-KO01040); (**B**) tryptophan metabolism (BIO-KO00380); (**C**) purine metabolism (BIO-KO00230); (**D**) thiamine metabolism (BIO-KO00730); (**E**) phenylalanine, tyrosine, and tryptophan metabolism (BIO-KO00400).

This study further analyzed the correlation between gut microbiome information and metabolite information using the BIO-Sankey network tool in MetOrigin (Figure 5). In tryptophan metabolism (KO00380), one genus (Bacteroides) was closely related to one reaction of two metabolites (indole, tryptophan). In purine metabolism (KO00230), five genera (*Blautia*, *Ruminococcus*, *Parabacteroides*, *Bacteroides,* and *Candidatus Arthromitus*) were closely related to seven reactions of two dysregulated metabolites (2-deoxyadenosine, uric acid). In thiamine metabolism (KO00730), three genera (*Ruminococcus*, *Blautia*, and *Bacteroides*) were closely related to three metabolic reactions of one metabolite (5-(2-hydroxyethyl)-4-methylthiazole), and one genus had a significant positive correlation with the metabolite (*p* < 0.05). In phenylalanine, tyrosine, and tryptophan biosynthesis (KO00400), four genera (*Blautia*, *Ruminococcus*, *Bacteroides*, and *Parabacteroides*) were closely related to two reactions of two metabolites (indole and tryptophan).

**Figure 6:**
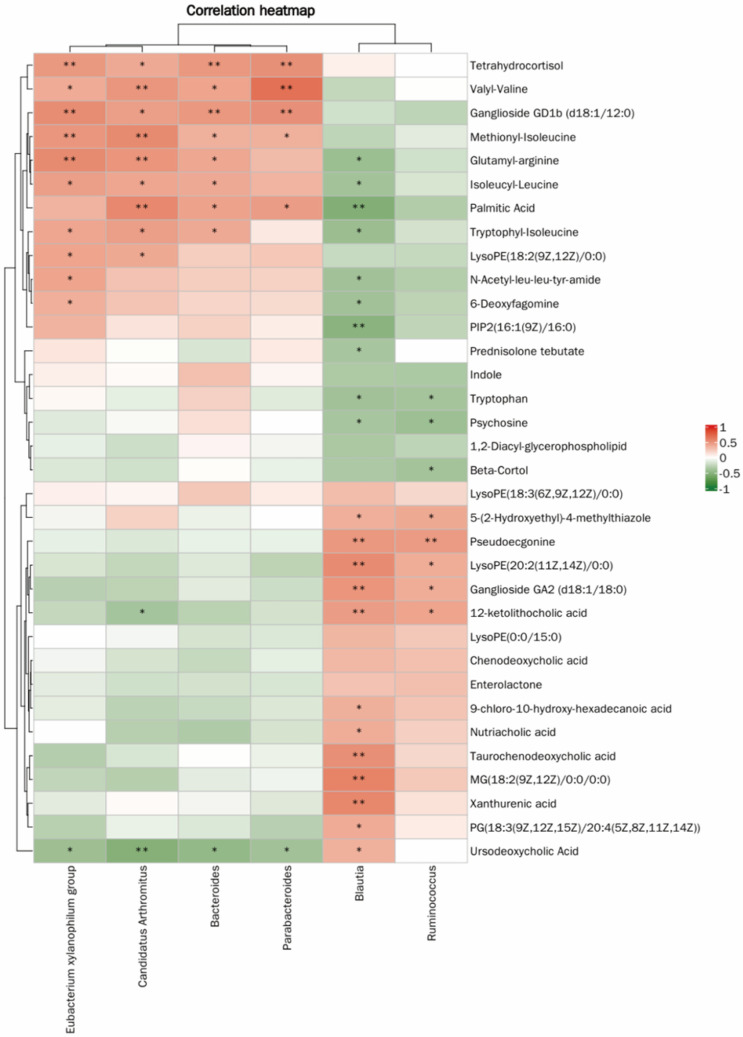
Heatmap of the correlation between microbial communities and metabolites. * *p* < 0.05, ** *p* < 0.01.

The correlation heatmap analysis further demonstrated a potential statistical correlation between gut microbiota and metabolites in immunosuppressed mice (Figure 6). The results indicated a close relationship between gut microbiota and metabolites, suggesting that JFP-Ps may improve the host’s immune status by regulating gut microbiota and their metabolic reactions.

## 4. Discussion

The intestinal microbiota, comprising a diverse consortium of symbiotic microorganisms inhabiting the gastrointestinal tract, is often regarded as an “invisible organ” of the host [25]. These symbiotic bacteria serve dual roles as both targets of host immunity and instigators of adaptive changes in host immune responses, playing significant roles in host physiology and immune regulation [26,27]. Recent studies have shown that the gut microbiota is a highly dynamic ecosystem shaped by host genetics, immune function, and diet, with its composition often reflecting the overall health status of the host [28]. Growing evidence also indicates that this microbial community acts as a key modulator of metabolic and immune-mediated diseases.

Cy is extensively utilized as a medication for the treatment of cancer. Nevertheless, at higher doses, Cy can disrupt gut microbiota and promote the proliferation of potential pathogenic bacteria [29]. Alterations in gut microbial composition in Cy-induced immunosuppressed mice treated with JFP-Ps were characterized using 16S rDNA sequencing. The increase in Shannon diversity was observed in the high-dose group, whereas richness indices (Chao1, ACE) remained unchanged, suggesting that the administration of high-dose JFP-Ps significantly enhanced the microbial community’s diversity and restored the gut microbiota’s phylum-level structure. In the gut microbiota of a healthy host, *Bacteroidetes* and *Firmicutes* represent the two most prevalent bacteria, collectively constituting over 90% of the gut microbiota. These results were in line with a previous report that Purple Red Rice Bran Anthocyanins (PRBAs) modulate the gut microbiota by enriching beneficial bacterial populations and enhancing the production of SCFAs [23]. Unexpectedly, an overexpansion of *Verrucomicrobiota* was observed in the intestines of Cy-induced immunosuppressed mice, accompanied by a pronounced decline in *Firmicutes* and *Bacteroidetes*, with an almost complete disturbance of the overall gut microbial architecture.

*Akkermansia* is a mucus-degrading bacterium that utilizes the mucus layer secreted by the intestinal epithelium as a carbon and nitrogen source for survival. It plays a role in maintaining intestinal barrier integrity, regulating host metabolism and immunity, and balancing the gut microbiota structure. However, during intestinal damage, the resulting gut dysbiosis and compromised barrier function create conditions conducive to *Akkermansia* overgrowth [30,31,32]. The higher efficiency of *Akkermansia* in utilizing mucin compared to other microbiota was a key factor contributing to its overgrowth. Cy may have eliminated or inhibited other commensal bacteria, thereby disrupting the balance of the intestinal ecosystem. LEfSe analysis revealed that *Akkermansia*, a representative member of *Verrucomicrobiota*, served as a phenotypic biomarker of the MC group, accounting for 71% of the microbial community. Excessive *Akkermansia* in the MC group may influence direct exposure of the intestinal surface to harmful pathogens. However, the effect of the decreased abundance of *Akkermansia* caused by JFP-Ps on the intestinal mucosal barrier needs further research.

Plant polysaccharides are functional ingredients indigestible by the host and beneficially modulate the gut microbiota. Previous studies have shown that polysaccharide interventions promote the expansion of beneficial bacterial populations while reducing the abundance of potentially pathogenic taxa [33,34]. Commensal probiotics residing in the intestine can enhance host immune defense by modulating and activating immune responses [35]. Plant polysaccharides can augment host immunity by targeting the gut microbiota. *Lactobacillus*, one of the most widely recognized probiotics in a healthy host’s gut, has been reported to be an enhancer of intestinal mucosal immunity [36]. Specifically, *Lactobacillus* improves the phagocytic function of macrophages, stimulates the proliferation of lymphocytes, enhances the cytotoxicity of natural killer cells, and regulates cytokine secretion [37,38]. An increase in *Lactobacillus* was found to promote the secretion of mucosal SIgA and elevate serum IgM levels, thereby reinforcing the integrity of the intestinal barrier and maintaining immunological balance in the host [39,40]. Additionally, *Lactobacillus* can upregulate the expression of the MUC3 gene to increase mucus production in intestinal goblet cells [38]. Our research showed that the ameliorative effects of JFP-Ps on the intestines of immunosuppressed mice appeared to be linked to an increase in the abundance of *Lactobacillus*.

*Lachnospiraceae*, the most abundant family in *Firmicutes*, is considered to be a beneficial bacterium that is closely related to host gut health. Known as active plant polysaccharide degraders, *Lachnospiraceae* have exhibited remarkable specificity in breaking down plant materials, including cellulose and hemicellulose [41,42]. Sun et al. [43] have demonstrated the bacteria’s ability to degrade non-starch polysaccharides, leading to the production of SCFAs and antimicrobial peptides. These substances can effectively inhibit pathogen colonization and induce anti-inflammatory responses in regulatory T-cells [44,45]. *Blautia* was found to alleviate intestinal inflammation and promote the production of SCFAs, particularly acetate [46,47]. Moreover, *Blautia* significantly contributes to recovery from pathogenic infections through modulation of endogenous amino acid metabolism, with its abundance markedly increasing during the recovery phase [48]. *Clostridiales* also belongs to one lineage within *Firmicutes*, acts as an obligate anaerobe that forms spores, and has been found to be immunogenic, effectively inducing the differentiation of regulatory T cells in the colon [49]. *Clostridiaceae* and *Enterobacteriaceae* are selectively stimulated by glucose and can absorb carbohydrates within the intestinal lumen [50]. Djukovic et al. [51] found that an increase in *Clostridiales* elevated the levels of butyrate and inhibited the proliferation of potentially harmful bacteria, such as *Enterobacteriaceae*, by reducing their nutrient sources. According to Bolotin et al. [52], Candidatus *Arthromitus* is considered essential for the postnatal activation of innate and adaptive immune responses within the murine intestinal tract. Conversely, it has been reported that the abundance of *Clostridiaceae* increases in individuals with inflammatory bowel disease, suggesting its potential pathogenic role [53]. The present investigation found that a high intake of JFP-Ps significantly increased the beneficial bacterium abundance in the *Firmicutes* family.

Additionally, *Muribaculaceae*, *Ruminococcaceae*, and *Rikenella* are bacteria capable of synthesizing SCFAs. It has been reported that *Muribaculaceae*, a core family of Bacteroidetes, produced propionate through fermentation and was linked to gut health and longevity in murine models [41,54]. *Ruminococcaceae*, which colonizes the cecum and colon, contributes to the degradation of dietary polysaccharides and fiber, producing butyrate with anti-inflammatory properties [55]. *Rikenella* is capable of fermenting propionate to generate energy in normal cells and stimulate gluconeogenesis [56]. Previous studies have suggested that the *Rikenellaceae_RC9_gut_group* and *Alistipes* may act as potential contributors to maintaining intestinal health and alleviating intestinal disorders [57,58]. In the present study, the abundance of *Alistipes*, *norank_f__Ruminococcaceae*, *Rikenellaceae_RC9_gut_group*, *norank_f__Muribaculaceae*, and *Rikenella* in the JFP-Ps group was largely restored. These findings are consistent with the levels of SCFAs detected in fecal samples. Overall, the study may suggest that JFP-Ps enhanced intestinal homeostasis by altering the gut microbiota composition, increasing beneficial bacterial populations and promoting SCFA synthesis in the intestines.

Metabolites produced by intestinal microorganisms act as key signaling molecules that mediate bidirectional crosstalk between the gut microbiota and the host. UPLC–Q-TOF–MS-based metabolomic profiling of mouse feces was conducted to clarify how JFP-Ps regulate gut microbial metabolism. MPEA revealed that six metabolic pathways were significantly enriched, including primary bile acid biosynthesis, thiamine metabolism, biosynthesis of the amino acids (phenylalanine, tyrosine, and tryptophan), unsaturated fatty acid biosynthesis, tryptophan metabolism, and purine metabolism. Among these, JFP-Ps markedly modulated host bile acid metabolism. In mammals, bile acids are generated in the liver from cholesterol, and their synthesis and signaling are largely governed by the nuclear farnesoid X receptor (FXR) and the membrane G protein-coupled bile acid receptor 1 (TGR5). These receptors are key regulators of energy metabolism and immune function [59]. While bile acids originate from the host, their metabolism requires the participation of the intestinal microbiota. Bacterial metabolism dictates the constitution of the enteric bile acid pool [60,61]. Consequently, alterations in gut microbiota structure can modulate host bile acid metabolism under pathological conditions. In a dextran sulfate sodium (DSS)-induced colitis model, lithocholic acid and ursodeoxycholic acid (UDCA) were found to alleviate intestinal inflammation, limit epithelial cell apoptosis, and sustain gastrointestinal barrier homeostasis [62]. Dietary supplementation of mice with chenodeoxycholic acid (CDCA) enhanced the expression of the mucin protein 2 (MUC2) gene in the ileum and the production of Paneth cell α-defensin, resulting in an elevated proportion of IgGκ+ B cells [63]. Among all the bile acids tested by Yang et al. [64], CDCA exhibited the strongest antibacterial activity and could target HilD to exert its anti-infective function. According to reports, isoursodeoxycholic acid (isoUDCA) can undergo extensive isomerization, such as being potentially converted to UDCA through intestinal and hepatic enzymes. Therefore, isoUDCA is considered to have pro-drug characteristics [65]. Additionally, bile acids serve an immunoregulatory function by directing the recruitment and differentiation of diverse immune cells. In mice receiving CDCA, the recruitment of monocytes, macrophages, and neutrophils into the intestinal mucosa was reduced, while the proportion of B cells increased during Salmonella typhimurium and Citrobacter rodentium infections [63]. The significance of bile acid regulation is also manifested in protecting the host from pathogenic infections. By converting host-derived primary bile acids into secondary bile acids through microbial metabolism, commensal bacteria help maintain colonization resistance against *Clostridium difficile* [60]. As a result, crosstalk between the gut microbiota and bile acids exerts profound effects on host physiology, modulating intestinal barrier integrity, innate and adaptive immune responses, and resistance to pathogen colonization. In this study, JFP-Ps reversed the levels of isoUDCA, 12-ketolithocholic acid, taurallocholic acid (TLCA), and CDCA, while significantly increasing the levels of taurochenodeoxycholic acid and UDCA. Given the potential role of these co-metabolites, particularly bile acid metabolites, in gut homeostasis, intestinal immunity, and gut immune homeostasis, JFP-Ps may modulate intestinal immune function through interactions between bile acids and the gut microbiota.

Dihomo-γ-linolenic acid (DGLA) is another key biomarker of the biosynthetic pathway of unsaturated fatty acids. DGLA is considered an important molecule in distinguishing between healthy and inflammatory states, as it is situated at a critical point in the metabolic pathway for producing anti-inflammatory derivatives or converting to pro-inflammatory lipid mediators through arachidonic acid (ARA). The relative ratio of ARA to DGLA is considered an important determinant in regulating inflammatory responses in the body. Certain disease states are characterized by abnormal fluctuations (either an increase or a decrease) in DGLA levels [66]. This study highlights an increase in DGLA content in the Cy group, which was decreased by JFP-Ps-H, indicating that JFP-Ps may exert an anti-inflammatory effect by inhibiting DGLA production.

In terms of biological evolution, amino acid metabolic pathways represent key checkpoints in immune function. The immune regulatory functions associated with these pathways rely on the consumption of specific amino acids and the generation of bioactive metabolites within the microenvironment [67]. Tryptophan is an essential aromatic amino acid that serves as a substrate for selected gut microbial populations. Tryptophan and its derivatives exert diverse biological activities, including the modulation of inflammatory processes, immune function, and host metabolism. In mammals, tryptophan catabolism occurs mainly via three pathways: the kynurenine pathway, the serotonin pathway, and a microbiota-dependent indole-producing pathway [68]. Reports have indicated that tryptophan levels in the feces of DSS-induced colitis mice were considerably elevated compared to those of normal individuals, indicating a close relationship between tryptophan metabolism and intestinal inflammation [69]. In this study, JFP-Ps may influence the levels of fecal tryptophan and indole and simultaneously increase levels of tryptophan metabolites, namely indoleacrylic acid and xanthurenic acid. Xanthurenic acid arises from tryptophan metabolism via the kynurenine pathway, whereas indoleacrylic acid is an indole derivative generated from tryptophan by the gut microbiota. Both compounds act as ligands of the aryl hydrocarbon receptor (AhR) and enhance intestinal barrier integrity by activating AhR and promoting IL-22 expression. [69,70,71]. Collectively, these findings indicate that JFP-Ps may stimulate tryptophan metabolism by reducing intestinal levels of aromatic amino acids and increasing the production of xanthurenic acid and indole propionic acid. This process is likely to facilitate regeneration of the intestinal epithelium and re-establishment of mucosal immune homeostasis.

The treatment with Cy also caused alterations in glycine-associated purine metabolic pathways. Purines have various cellular functions, including their role as building blocks of DNA and RNA, as well as providing a source of energy [71]. Uric acid serves as a biomarker for tissue damage, kidney impairment, gout, and intestinal barrier damage [72]. In the present work, administration of JFP-Ps resulted in a reduction of uric acid levels and elevation of 2′-deoxyadenosine concentrations, thereby demonstrating the beneficial actions in Cy-induced immunosuppressed mice. Microbial metabolites serve as critical mediators in the gut microbiota–host interaction. Spearman correlation-based heatmap analysis revealed significant statistical correlations at the genus level between 50 distinct metabolites and six microbial genera: *Candidatus_Arthromitus*, *Eubacterium xylanophilum group*, *Bacteroides*, *Parabacteroides*, *Ruminococcus*, and *Blautia*. In summary, JFP-Ps modulate numerous physiological processes by affecting the composition of the gut microbiota and the metabolism of small molecules. Nevertheless, additional studies are necessary to clarify the regulatory mechanisms underlying these effects.

## 5. Limitations

This study has several limitations. First, although the sample size (*n* = 6) is consistent with similar studies, it remains small for detecting effect sizes. Second, while changes in mucin-degrading bacteria were observed, the lack of mucin quantification means that more direct evidence for alterations in barrier integrity is unavailable. The absence of histological analysis of intestinal tissues and cytokine profiling limits a more comprehensive assessment of immuno-modulatory effects at both tissue and systemic levels. Third, the research group’s previous characterization data were referenced, and the endotoxin level of the specific batch of JFP-Ps used in this animal study was not reconfirmed. Finally, the observed correlations between specific microbial changes and metabolite alterations, while biologically plausible, are associative and do not establish a mechanistic causal relationship. Future studies with larger cohorts integrating multi-omics approaches and mechanistic models are needed to validate and extend these findings.

## 6. Conclusions

This study examined the immuno-modulatory effects of JFP-Ps in Cy-induced immunosuppressed mice. The results showed that a high dose of JFP-Ps significantly improved the microbiota and metabolic profile of Cy-treated mice. Specifically, JFP-Ps intervention promoted the proliferation of probiotic bacteria, such as *Lactobacillus*, *Lachnospiraceae*, *norank_f__Muribaculaceae*, *Rikenellaceae_RC9_gut_group*, *Alistipes*, *norank_f__Ruminococcaceae*, and *Rikenella*, while reducing the abundance of some other bacteria, like *Akkermansia*. Moreover, metabolomics analysis revealed that JFP-Ps improved the fecal metabolic profile by modulating bile acid, amino acid, and fatty acid metabolism. Spearman correlation analysis revealed a robust link between gut microbiota composition and alterations in microbial metabolites, suggesting that JFP-Ps may ameliorate Cy-induced intestinal damage through regulation of the gut microbiota structure and its co-metabolism with the host. These findings offer significant insights into the immuno-modulatory actions of JFP-Ps within the context of the host–microbiota metabolic axis. In the future, our subsequent studies will focus on exploring the molecular mechanisms of JFP-Ps on intestinal injury through histological and immunological approaches.

## Figures and Tables

**Table 1:** The concentration of acetic, propionic, i-butyric, n-butyric, i-valeric acid, nvaleric, and total SCFAs in different groups.

	NC	MC	JFP-Ps-L	JFP-Ps-M	JFP-Ps-H
Acetic acid (mg/g)	5.79 ± 0.65 ^c^	2.42 ± 0.19 ^a^	2.44 ± 0.33 ^a^	3.90 ± 0.46 ^b^	3.81 ± 0.47 ^b^
Propionic acid (mg/g)	2.03 ± 0.22 ^b^	1.12 ± 0.09 ^a^	1.31 ± 0.11 ^a^	1.88 ± 0.19 ^b^	2.06 ± 0.13 ^b^
i-Butyric acid (mg/g)	0.14 ± 0.01 ^c^	0.09 ± 0.00 ^a^	0.11 ± 0.01 ^ab^	0.13 ± 0.01 ^bc^	0.18 ± 0.01 ^d^
n-Butyric acid (mg/g)	2.29 ± 0.39 ^c^	0.83 ± 0.06 ^a^	1.05 ± 0.06 ^ab^	1.21 ± 0.13 ^ab^	1.66 ± 0.17 ^b^
i-Valeric acid (mg/g)	0.15 ± 0.01 ^b^	0.10 ± 0.01 ^a^	0.12 ± 0.02 ^ab^	0.14 ± 0.01 ^ab^	0.23 ± 0.02 ^c^
n-Valeric acid (mg/g)	0.16 ± 0.01 ^c^	0.06 ± 0.01 ^a^	0.10 ± 0.02 ^b^	0.10 ± 0.01 ^b^	0.19 ± 0.01 ^c^
Total SCFAs (mg/g)	10.57 ± 1.06 ^c^	4.61 ± 0.27 ^a^	5.13 ± 0.92 ^a^	7.34 ± 0.60 ^b^	8.14 ± 0.66 ^b^

The data are expressed as mean ± standard error of the mean (SEM), *n* = 6. Significant differences are denoted by distinct letters based on Duncan’s multiple range test at a significance level of *p* < 0.05.

## Data Availability

The original contributions presented in the study are included in the article/Supplementary Materials. Further inquiries can be directed to the corresponding authors.

## Supplementary Materials

The following supporting information can be downloaded at https://www.mdpi.com/article/10.3390/foods15010138/s1: Figure S1: Assessment analysis of the intestinal flora from each sample. (A) Rarefaction curves of Sobs index of intestinal flora at the OTU level; (B) Rarefaction curves of Shannon index of intestinal flora at the OTU level. Figure S2: Typical total ion flow chromatogram of fecal samples in positive ion mode (A) and negative ion mode (B). Figure S3: OPLS-DA scores of mouse fecal samples. (A,C,E,G) are positive ion modes; (B,D,F,H) are negative ion modes. Figure S4: The permutation of OPLS-DA (A,C,E,G) in positive ion mode and (B,D,F,H) in negative ion mode in fecal samples of mice. Figure S5: Volcano plot of differential metabolites. (A,C,E,G) are positive ion modes; (B,D,F,H) are negative ion modes. Table S1: Fecal differential metabolites in the NC, MC, and JFP-Ps-H groups.

## Abbreviations

JFP-Ps, *Artocarpus heterophyllus* Lam. (jackfruit) polysaccharide; Cy, cyclophosphamide; SCFAs, short-chain fatty acids; SPF, specific pathogen-free; BW, body weight; GC, gas chromatography; PCR, polymerase chain reaction; SEM, standard error of the mean; OTU, operational taxonomic unit; PCA, principal coordinates analysis; PRBA, Purple Red Rice Bran Anthocyanins; MPEA, metabolic pathway enrichment analysis; FXR, farnesoid X receptor; TGR5, G protein-coupled bile acid receptor 1; DSS, dextran sulfate sodium; UDCA, ursodeoxycholic acid; CDCA, chenodeoxycholic acid; MUC2, mucin protein 2; isoUDCA, isoursodeoxycholic acid; TLCA, taurallocholic acid; DGLA, Dihomo-γ-linolenic acid; ARA, arachidonic acid; AhR, aryl hydrocarbon receptor.

## References

[1] Lavelle A., Sokol H. (2020). Gut microbiota-derived metabolites as key actors in inflammatory bowel disease. Nat. Rev. Gastroenterol. Hepatol..

[2] Nicholson J.K., Holmes E., Kinross J., Burcelin R., Gibson G., Jia W., Pettersson S. (2012). Host-gut microbiota metabolic interactions. Science.

[3] Xi M., Tang H., Zhang Y., Ge W., Chen Y., Cui X. (2021). Microbiome-metabolomic analyses of the impacts of dietary stachyose on fecal microbiota and metabolites in infants intestinal microbiota-associated mice. J. Sci. Food Agric..

[4] Jiang Z., He L., Li D., Zhuo L., Chen L., Shi R.-Q., Luo J., Feng Y., Liang Y., Li D. (2025). Human gut microbial aromatic amino acid and related metabolites prevent obesity through intestinal immune control. Nat. Metab..

[5] Araújo J.R., Marques C., Rodrigues C., Calhau C., Faria A. (2024). The metabolic and endocrine impact of diet-derived gut microbiota metabolites on ageing and longevity. Ageing Res. Rev..

[6] Mann E.R., Lam Y.K., Uhlig H.H. (2024). Short-chain fatty acids: Linking diet, the microbiome and immunity. Nat. Rev. Immunol..

[7] Won T.H., Arifuzzaman M., Parkhurst C.N., Miranda I.C., Zhang B., Hu E., Kashyap S., Letourneau J., Jin W.B., Fu Y. (2025). Host metabolism balances microbial regulation of bile acid signalling. Nature.

[8] Louis P., Flint H.J. (2017). Formation of propionate and butyrate by the human colonic microbiota. Environ. Microbiol..

[9] Wu Y., Zhu C., Zhang Y., Li Y., Sun J. (2019). Immunomodulatory and antioxidant effects of pomegranate peel polysaccharides on immunosuppressed mice. Int. J. Biol. Macromol..

[10] de Jonge M.E., Huitema A.D.R., Rodenhuis S., Beijnen J.H. (2005). Clinical Pharmacokinetics of Cyclophosphamide. Clin. Pharmacokinet..

[11] Kumar V.P., Venkatesh Y.P. (2016). Alleviation of cyclophosphamide-induced immunosuppression in Wistar rats by onion lectin (*Allium cepa* agglutinin). J. Ethnopharmacol..

[12] Ying M., Yu Q., Zheng B., Wang H., Wang J., Chen S., Nie S., Xie M. (2020). Cultured *Cordyceps sinensis* polysaccharides modulate intestinal mucosal immunity and gut microbiota in cyclophosphamide-treated mice. Carbohydr. Polym..

[13] Shafiq M., Mehmood S., Yasmin A., Khan S.J., Khan N.H., Ali S. (2017). Evaluation of phytochemical, nutritional and antioxidant activity of indigenously grown jackfruit (*Artocarpus heterophyllus* Lam). J. Sci. Res..

[14] Gupta A., Marquess A.R., Pandey A.K., Bishayee A. (2023). Jackfruit (*Artocarpus heterophyllus* Lam.) in health and disease: A critical review. Crit. Rev. Food Sci. Nutr..

[15] Tan Y.F., Li H.L., Lai W.Y., Zhang J.Q. (2013). Crude dietary polysaccharide fraction isolated from jackfruit enhances immune system activity in mice. J. Med. Food.

[16] Kaur J., Singh Z., Shah H.M.S., Mazhar M.S., Hasan M.U., Woodward A. (2024). Insights into phytonutrient profile and postharvest quality management of jackfruit: A review. Crit. Rev. Food Sci. Nutr..

[17] Fu Y., Guo J., Xie Y., Yu X., Su Q., Qiang L., Kong L., Liu Y. (2020). Prenylated chromones from the fruits of *Artocarpus heterophyllus* and their potential anti-HIV-1 activities. J. Agric. Food. Chem..

[18] Sun G., Zheng Z., Lee M.-H., Xu Y., Kang S., Dong Z., Wang M., Gu Z., Li H., Chen W. (2017). Chemoprevention of colorectal cancer by Artocarpin, a dietary phytochemical from *Artocarpus heterophyllus*. J. Agric. Food. Chem..

[19] Zhu K., Zhang Y., Nie S., Xu F., He S., Gong D., Wu G., Tan L. (2017). Physicochemical properties and in vitro antioxidant activities of polysaccharide from *Artocarpus heterophyllus* Lam. pulp. Carbohydr. Polym..

[20] Wiater A., Paduch R., Trojnar S., Choma A., Pleszczyńska M., Adamczyk P., Pięt M., Próchniak K., Szczodrak J., Strawa J. (2020). The effect of water-soluble polysaccharide from jackfruit (*Artocarpus heterophyllus* Lam.) on human colon carcinoma cells cultured in vitro. Plants.

[21] Zeng S., Cao J., Chen Y., Li C., Wu G., Zhu K., Chen X., Xu F., Liu Q., Tan L. (2022). Polysaccharides from *Artocarpus heterophyllus* Lam. (jackfruit) pulp improves intestinal barrier functions of high fat diet-induced obese rats. Front. Nutr..

[22] Zeng S., Cao J., Wei C., Chen Y., Liu Q., Li C., Zhang Y., Zhu K., Wu G., Tan L. (2023). Polysaccharides from *Artocarpus heterophyllus* Lam. (jackfruit) pulp alleviate obesity by modulating gut microbiota in high fat diet-induced rats. Food Hydrocoll..

[23] Chen T., Shen M., Yu Q., Chen Y., Wen H., Lu H., Chen S., Xie J. (2022). Purple red rice anthocyanins alleviate intestinal damage in cyclophosphamide-induced mice associated with modulation of intestinal barrier function and gut microbiota. Food Chem..

[24] Zhu K., Fan H., Zeng S., Nie S., Zhang Y., Tan L., Li C., Xu F., Liu Q., Wu G. (2021). Polysaccharide from *Artocarpus heterophyllus* Lam. (jackfruit) pulp modulates gut microbiota composition and improves short-chain fatty acids production. Food Chem..

[25] Ross F.C., Patangia D., Grimaud G., Lavelle A., Dempsey E.M., Ross R.P., Stanton C. (2024). The interplay between diet and the gut microbiome: Implications for health and disease. Nat. Rev. Microbiol..

[26] Yang L., Liu S., Ding J., Dai R., He C., Xu K., Honaker C.F., Zhang Y., Siegel P., Meng H. (2017). Gut microbiota co-microevolution with selection for host humoral immunity. Front. Microbiol..

[27] Simpson R.C., Shanahan E.R., Scolyer R.A., Long G.V. (2023). Towards modulating the gut microbiota to enhance the efficacy of immune-checkpoint inhibitors. Nat. Rev. Clin. Oncol..

[28] Ahmed I., Roy B.C., Khan S.A., Septer S., Umar S. (2016). Microbiome, metabolome and inflammatory bowel disease. Microorganisms.

[29] Xu D.H., Xie H.Y., Li Y.L., Wang L., Qing L., Yu S., Zhao J.L., Zeng H. (2024). Phyllanthus emblica polysaccharide (PEP) attenuates cyclophosphamide-induced immunosuppression through microbiota-dependent or –independent regulation of immune responses. J. Funct. Foods.

[30] Bae M., Cassilly C.D., Liu X., Park S.-M., Tusi B.K., Chen X., Kwon J., Filipčík P., Bolze A.S., Liu Z. (2022). Akkermansia muciniphila phospholipid induces homeostatic immune responses. Nature.

[31] Khan S., Waliullah S., Godfrey V., Khan M.A.W., Ramachandran R.A., Cantarel B.L., Behrendt C., Peng L., Hooper L.V., Zaki H. (2020). Dietary simple sugars alter microbial ecology in the gut and promote colitis in mice. Sci. Transl. Med..

[32] Martin-Gallausiaux C., Garcia-Weber D., Lashermes A., Larraufie P., Marinelli L., Teixeira V., Rolland A., Béguet-Crespel F., Brochard V., Quatremare T. (2022). *Akkermansia* muciniphila upregulates genes involved in maintaining the intestinal barrier function via ADP-heptose-dependent activation of the ALPK1/TIFA pathway. Gut Microbes.

[33] Zhang C., Pi X., Li X., Huo J., Wang W. (2024). Edible herbal source-derived polysaccharides as potential prebiotics: Composition, structure, gut microbiota regulation, and its related health effects. Food Chem..

[34] Yang J., Lin J., Luo Y., Chen X., Shen M., Wang Y., Xie J. (2025). Nutritional intervention based on curcumin-loaded polysaccharides nanoparticle for synergetic improvement of ulcerative colitis. Food Sci. Hum. Wellness.

[35] Koren O., Konnikova L., Brodin P., Mysorekar I.U., Collado M.C. (2024). The maternal gut microbiome in pregnancy: Implications for the developing immune system. Nat. Rev. Gastroenterol. Hepatol..

[36] Ding Y., Yan Y., Chen D., Ran L., Mi J., Lu L., Jing B., Li X., Zeng X., Cao Y. (2019). Modulating effects of polysaccharides from the fruits of *Lycium barbarum* on the immune response and gut microbiota in cyclophosphamide-treated mice. Food Funct..

[37] Azad M.A.K., Sarker M., Wan D. (2018). Immunomodulatory effects of probiotics on cytokine profiles. BioMed Res. Int..

[38] Orel R., Kamhi Trop T. (2014). Intestinal microbiota, probiotics and prebiotics in inflammatory bowel disease. World J. Gastroenterol..

[39] Li Y., Guo B., Wu Z., Wang W., Li C., Liu G., Cai H. (2020). Effects of fermented soybean meal supplementation on the growth performance and cecal microbiota community of broiler chickens. Animals.

[40] Sun Y., Chen S., Wei R., Xie X., Wang C., Fan S., Zhang X., Su J., Liu J., Jia W. (2018). Metabolome and gut microbiota variation with long-term intake of *Panax ginseng* extracts on rats. Food Funct..

[41] Guo Z., Hu B., Zhu L., Yang Y., Liu C., Liu F., Shi Y., Li M., Gu Z., Xin Y. (2022). Microbiome-metabolomics insights into the feces of high-fat diet mice to reveal the anti-obesity effects of yak (*Bos grunniens*) bone collagen hydrolysates. Food Res. Int..

[42] Fan S., Ding Y., Hu Z., Zhang Z., Fu L., Zhang J., Zhu Y., Bai J., Xiao X. (2025). Inter-individual variation in human microbiota drives differential impacts on the fermentability of insoluble bran by soluble β-glucans from whole barley. Food Hydrocoll..

[43] Sun Y., Wang F., Liu Y., Liu S., An Y., Xue H., Wang J., Xia F., Chen X., Cao Y. (2022). Microbiome-metabolome responses of Fuzhuan brick tea crude polysaccharides with immune-protective benefit in cyclophosphamide-induced immunosuppressive mice. Food Res. Int..

[44] Gao Y., Liu Y., Ma F., Sun M., Song Y., Xu D., Mu G., Tuo Y. (2021). Lactobacillus plantarum Y44 alleviates oxidative stress by regulating gut microbiota and colonic barrier function in Balb/C mice with subcutaneous D-galactose injection. Food Funct..

[45] Sorbara M.T., Littmann E.R., Fontana E., Moody T.U., Kohout C.E., Gjonbalaj M., Eaton V., Seok R., Leiner I.M., Pamer E.G. (2020). Functional and genomic variation between human-derived isolates of *Lachnospiraceae* reveals inter-and intra-species diversity. Cell Host Microbe.

[46] Li H., Christman L.M., Li R., Gu L. (2020). Synergic interactions between polyphenols and gut microbiota in mitigating inflammatory bowel diseases. Food Funct..

[47] Louis P., Hold G.L., Flint H.J. (2014). The gut microbiota, bacterial metabolites and colorectal cancer. Nat. Rev. Microbiol..

[48] Lin H., Chen S., Shen L., Hu T., Cai J., Zhan S., Liang J., Huang M., Xian M., Wang S. (2022). Integrated analysis of the cecal microbiome and plasma metabolomics to explore NaoMaiTong and its potential role in changing the intestinal flora and their metabolites in ischemic stroke. Front. Pharmacol..

[49] Sargsian S., Mondragón-Palomino O., Lejeune A., Ercelen D., Jin W.-B., Varghese A., Lim Y.A.L., Guo C.-J., Loke P.n., Cadwell K. (2024). Functional characterization of helminth-associated Clostridiales reveals covariates of Treg differentiation. Microbiome.

[50] Wüst P.K., Horn M.A., Drake H.L. (2011). Clostridiaceae and Enterobacteriaceae as active fermenters in earthworm gut content. ISME J..

[51] Djukovic A., Garzón M.J., Canlet C., Cabral V., Lalaoui R., García-Garcerá M., Rechenberger J., Tremblay-Franco M., Peñaranda I., Puchades-Carrasco L. (2022). *Lactobacillus* supports *Clostridiales* to restrict gut colonization by multidrug-resistant Enterobacteriaceae. Nat. Commun..

[52] Bolotin A., De Wouters T., Schnupf P., Bouchier C., Loux V., Rhimi M., Jamet A., Dervyn R., Boudebbouze S., Blottière Hervé M. (2014). Genome sequence of “*Candidatus Arthromitus*” sp. strain SFB-mouse-NL, a commensal bacterium with a key role in postnatal maturation of gut immune functions. Genome Announc..

[53] Muñiz Pedrogo D.A., Chen J., Hillmann B., Jeraldo P., Al-Ghalith G., Taneja V., Davis J.M., Knights D., Nelson H., Faubion W.A. (2019). An increased abundance of *Clostridiaceae* characterizes arthritis in inflammatory bowel disease and rheumatoid arthritis: A cross-sectional study. Inflamm. Bowel Dis..

[54] Smith Byron J., Miller Richard A., Schmidt Thomas M. (2021). *Muribaculaceae* genomes assembled from metagenomes suggest genetic drivers of differential response to acarbose treatment in mice. mSphere.

[55] Sun Y., Liu Y., Ai C., Song S., Chen X. (2019). *Caulerpa lentillifera* polysaccharides enhance the immunostimulatory activity in immunosuppressed mice in correlation with modulating gut microbiota. Food Funct..

[56] Shi H., Chang Y., Gao Y., Wang X., Chen X., Wang Y., Xue C., Tang Q. (2017). Dietary fucoidan of *Acaudina molpadioides* alters gut microbiota and mitigates intestinal mucosal injury induced by cyclophosphamide. Food Funct..

[57] Han X., Bai B., Zhou Q., Niu J., Yuan J., Zhang H., Jia J., Zhao W., Chen H. (2020). Dietary supplementation with polysaccharides from *Ziziphus Jujuba* cv. *Pozao* intervenes in immune response via regulating peripheral immunity and intestinal barrier function in cyclophosphamide-induced mice. Food Funct..

[58] Zhou Z., He W., Tian H., Zhan P., Liu J. (2023). Thyme (*Thymus vulgaris* L.) polyphenols ameliorate DSS-induced ulcerative colitis of mice by mitigating intestinal barrier damage, regulating gut microbiota, and suppressing TLR4/NF-κB-NLRP3 inflammasome pathways. Food Funct..

[59] Li C., Jiao T., Liu W., Luo Y., Wang J., Guo X., Tong X., Lin Z., Sun C., Wang K. (2022). Hepatic cytochrome P450 8B1 and cholic acid potentiate intestinal epithelial injury in colitis by suppressing intestinal stem cell renewal. Cell Stem Cell.

[60] Larabi A.B., Masson H.L.P., Bäumler A.J. (2023). Bile acids as modulators of gut microbiota composition and function. Gut Microbes.

[61] Yang J., Chen X., Lin J., Shen M., Wang Y., Sarkar A., Wen H., Xie J. (2024). Co-delivery of resveratrol and curcumin based on *Mesona chinensis* polysaccharides/zein nanoparticle for targeted alleviation of ulcerative colitis. Food Biosci..

[62] Lajczak-McGinley N.K., Porru E., Fallon C.M., Smyth J., Curley C., McCarron P.A., Tambuwala M.M., Roda A., Keely S.J. (2020). The secondary bile acids, ursodeoxycholic acid and lithocholic acid, protect against intestinal inflammation by inhibition of epithelial apoptosis. Physiol. Rep..

[63] Tremblay S., Romain G., Roux M., Chen X.L., Brown K., Gibson Deanna L., Ramanathan S., Menendez A. (2017). Bile acid administration elicits an intestinal antimicrobial program and reduces the bacterial burden in two mouse models of enteric infection. Infect. Immun..

[64] Yang X., Stein K.R., Hang H.C. (2023). Anti-infective bile acids bind and inactivate a Salmonella virulence regulator. Nat. Chem. Biol..

[65] Marschall H.U., Broomé U., Einarsson C., Alvelius G., Thomas H.G., Matern S. (2001). Isoursodeoxycholic acid: Metabolism and therapeutic effects in primary biliary cirrhosis 1. J. Lipid Res..

[66] Mustonen A.M., Nieminen P. (2023). Dihomo-γ-linolenic acid (20:3n-6)-metabolism, derivatives, and potential significance in chronic inflammation. Int. J. Mol. Sci..

[67] Mondanelli G., Iacono A., Allegrucci M., Puccetti P., Grohmann U. (2019). Immunoregulatory interplay between Arginine and Tryptophan metabolism in health and disease. Front. Immunol..

[68] Xue C., Li G., Zheng Q., Gu X., Shi Q., Su Y., Chu Q., Yuan X., Bao Z., Lu J. (2023). Tryptophan metabolism in health and disease. Cell Metab..

[69] Yang W., Ren D., Zhao Y., Liu L., Yang X. (2021). Fuzhuan brick tea polysaccharide improved ulcerative colitis in association with gut microbiota-derived tryptophan metabolism. J. Agric. Food. Chem..

[70] Agus A., Planchais J., Sokol H. (2018). Gut microbiota regulation of tryptophan metabolism in health and disease. Cell Host Microbe.

[71] Zhou R., He D., Xie J., Zhou Q., Zeng H., Li H., Huang L. (2021). The synergistic effects of polysaccharides and ginsenosides from American ginseng (*Panax quinquefolius* L.) ameliorating cyclophosphamide-induced intestinal immune disorders and gut barrier dysfunctions based on microbiome-metabolomics analysis. Front. Immunol..

[72] Tian H., Liang Y., Liu G., Li Y., Deng M., Liu D., Guo Y., Sun B. (2021). Moringa oleifera polysaccharides regulates caecal microbiota and small intestinal metabolic profile in C57BL/6 mice. Int. J. Biol. Macromol..

