# Supplementary material for: Polysaccharide from Artocarpus heterophyllus Lam. Pulp Ameliorates Cyclophosphamide-Induced Intestinal Damage by Regulating Gut Microbiota and Co-Metabolites
Source: Foods. 2026 Jan 2;15(1):138. doi: 10.3390/foods15010138 (PMC12785341; doi:10.3390/foods15010138)
Supplement: Supplementary file 1 [file foods-15-00138-s001.zip › foods-4014053-supplementary.pdf]

Supplementary Documents

Polysaccharide from *Artocarpus heterophyllus* Lam. Pulp ameliorates cyclophosphamide-induced intestinal damage in mice via the gut microbiota and metabolites alteration

Figure S1 Assessment analysis of the intestinal flora from each sample. (A) Rarefaction curves of Sobs index of intestinal flora at the OTU level, (B) Rarefaction curves of Shannon index of intestinal flora at the OTU level.

Figure S2 Typical total ion flow chromatogram of fecal samples in positive ion mode (A) and negative ion mode(B)

Figure S3 OPLS-DA scores of mice fecal samples. (A), (C), (E) and (G) are positive ion modes; (B), (D), (F), and (H) are the negative ion modes.

Figure S4 The permutation of OPLS-DA (A), (C), (E), (G) in positive ion mode and (B), (D), (F), (H) in negative ion mode in fecal samples of mice.

Figure S5 Volcano plot of differential metabolites. (A), (C), (E) and (G) are positive ion modes. (B), (D), (F), and (H) are the negative ion mode

Table S1 Fecal differential metabolites in NC, MC, and JFP-Ps-H groups

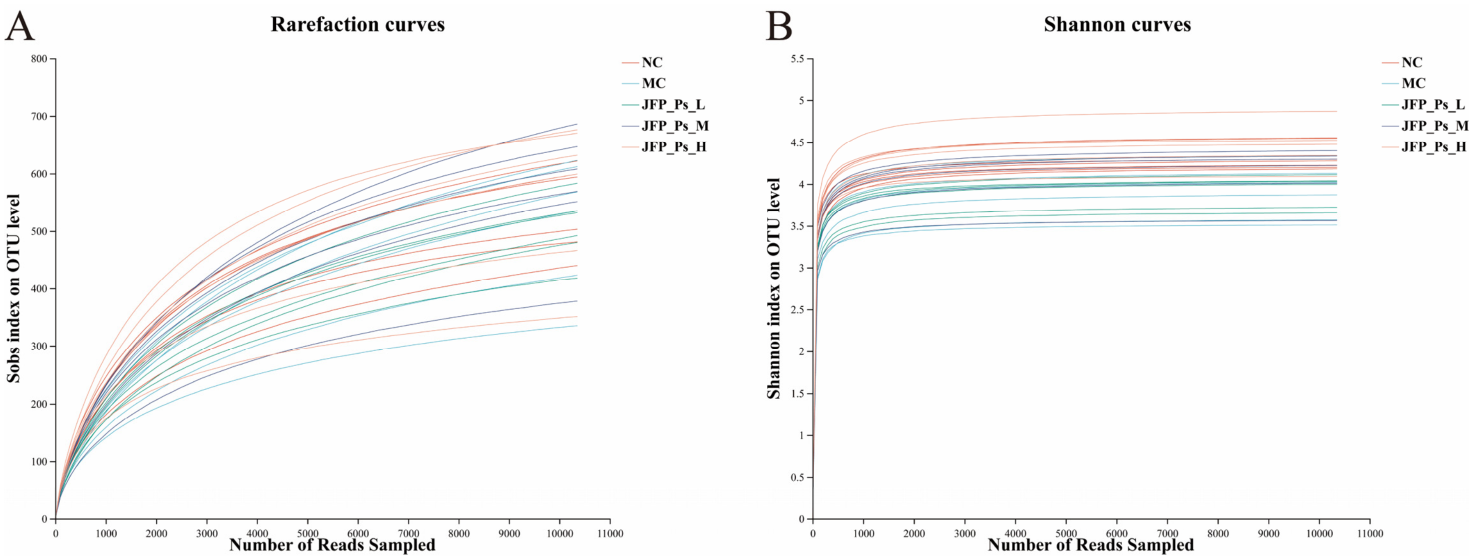

Figure S1 Assessment analysis of the intestinal flora from each sample

(A) Rarefaction curves of Sobs index of intestinal flora at the OTU level, (B) Rarefaction curves of Shannon index of intestinal flora at the OTU level.

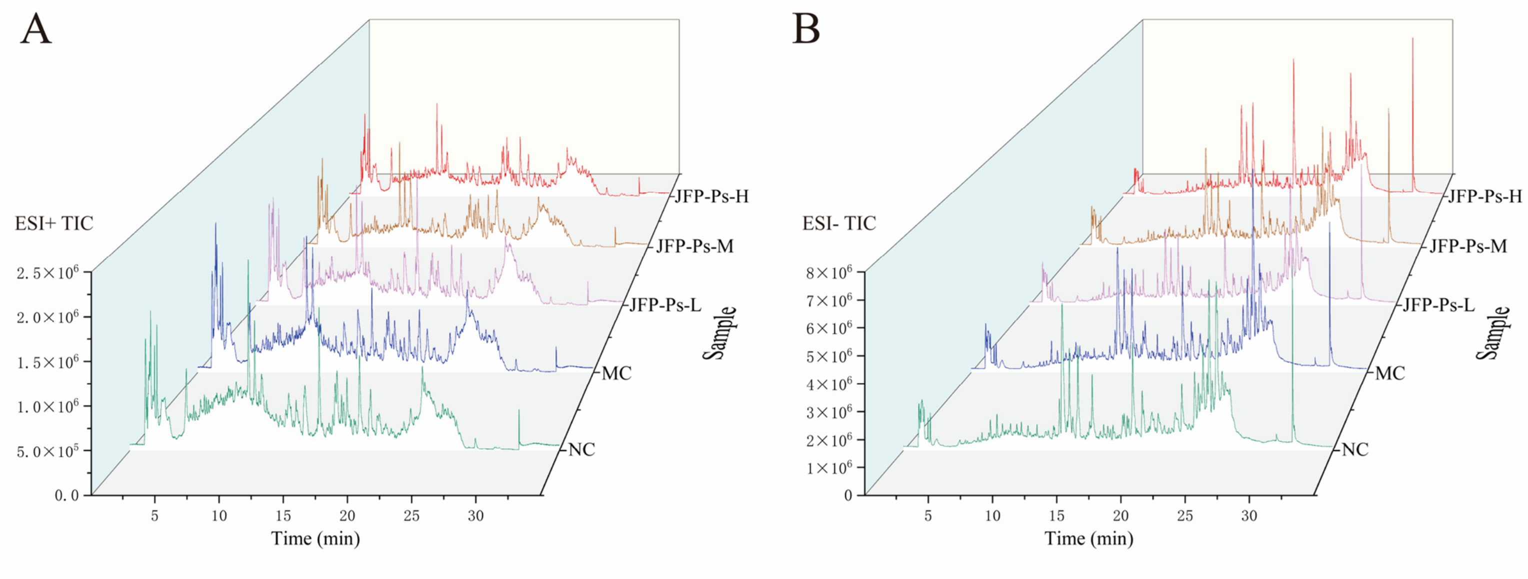

Figure S2 Typical total ion flow chromatogram of fecal samples in positive ion mode (A) and negative ion mode(B)

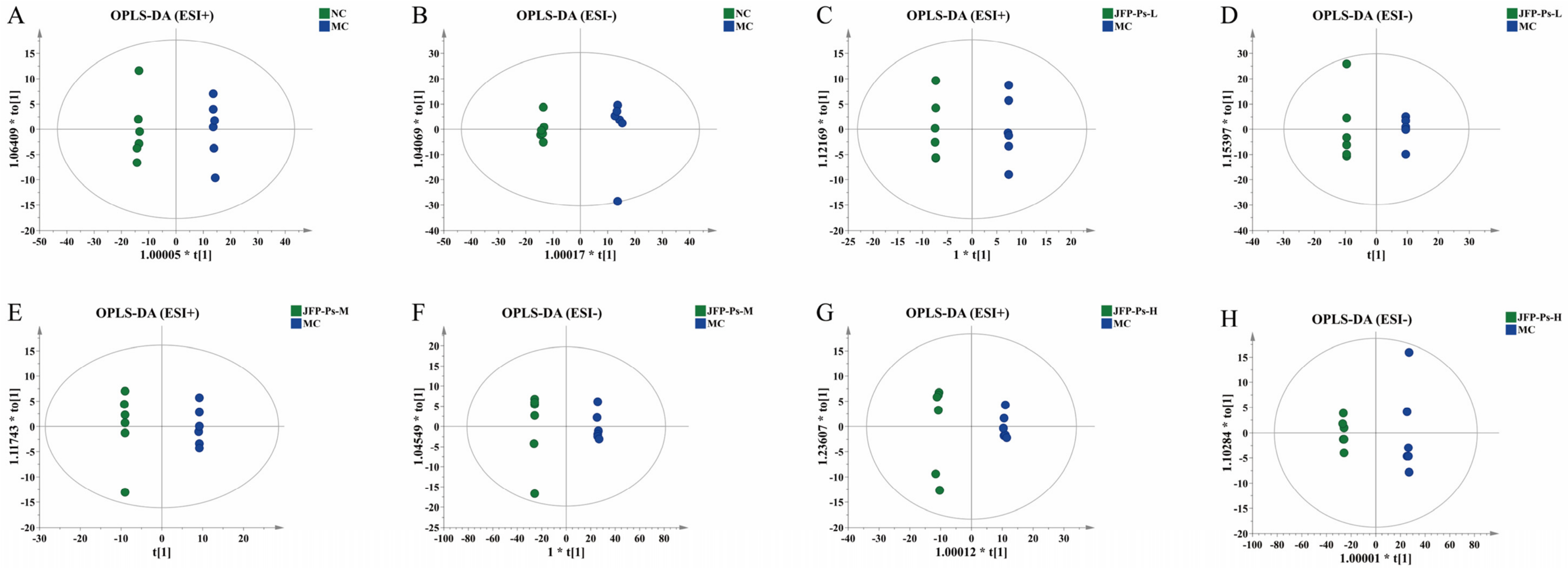

Figure S3 OPLS-DA scores of mice fecal samples. (A), (C), (E) and (G) are positive ion modes; (B), (D), (F), and (H) are the negative ion modes.

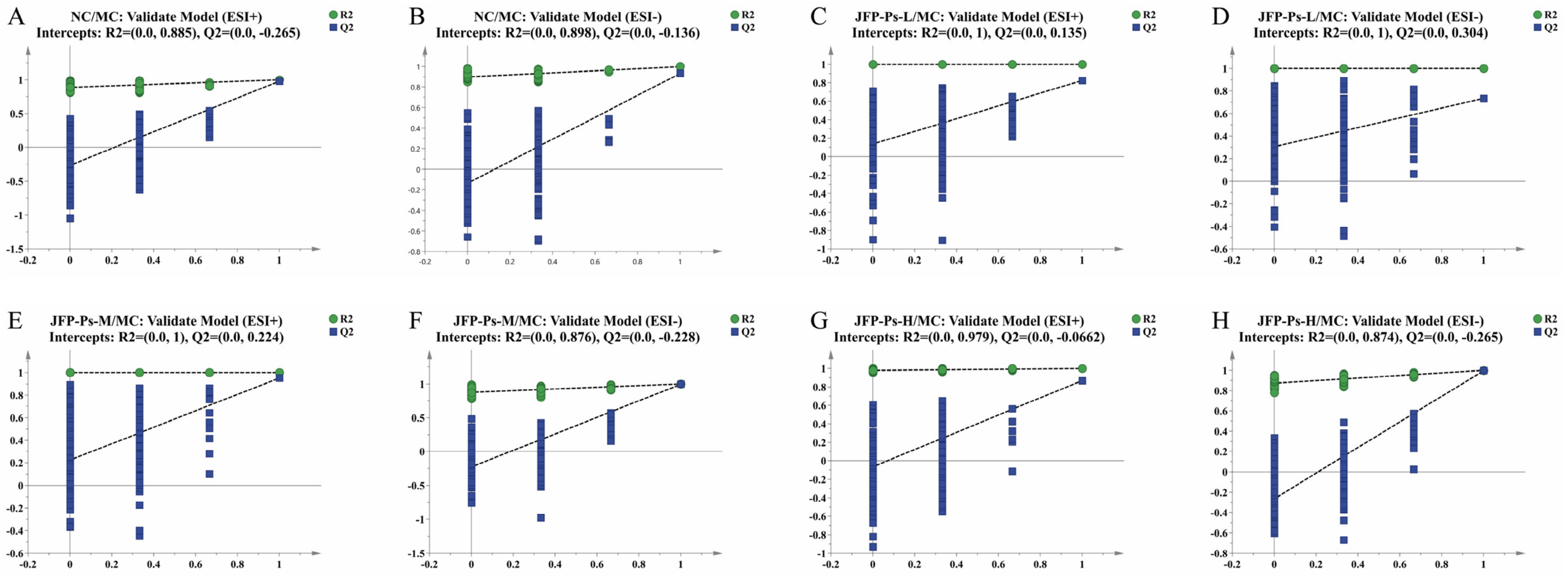

Figure S4 The permutation of OPLS-DA (A), (C), (E), (G) in positive ion mode and (B), (D), (F), (H) in negative ion mode in fecal samples of mice.

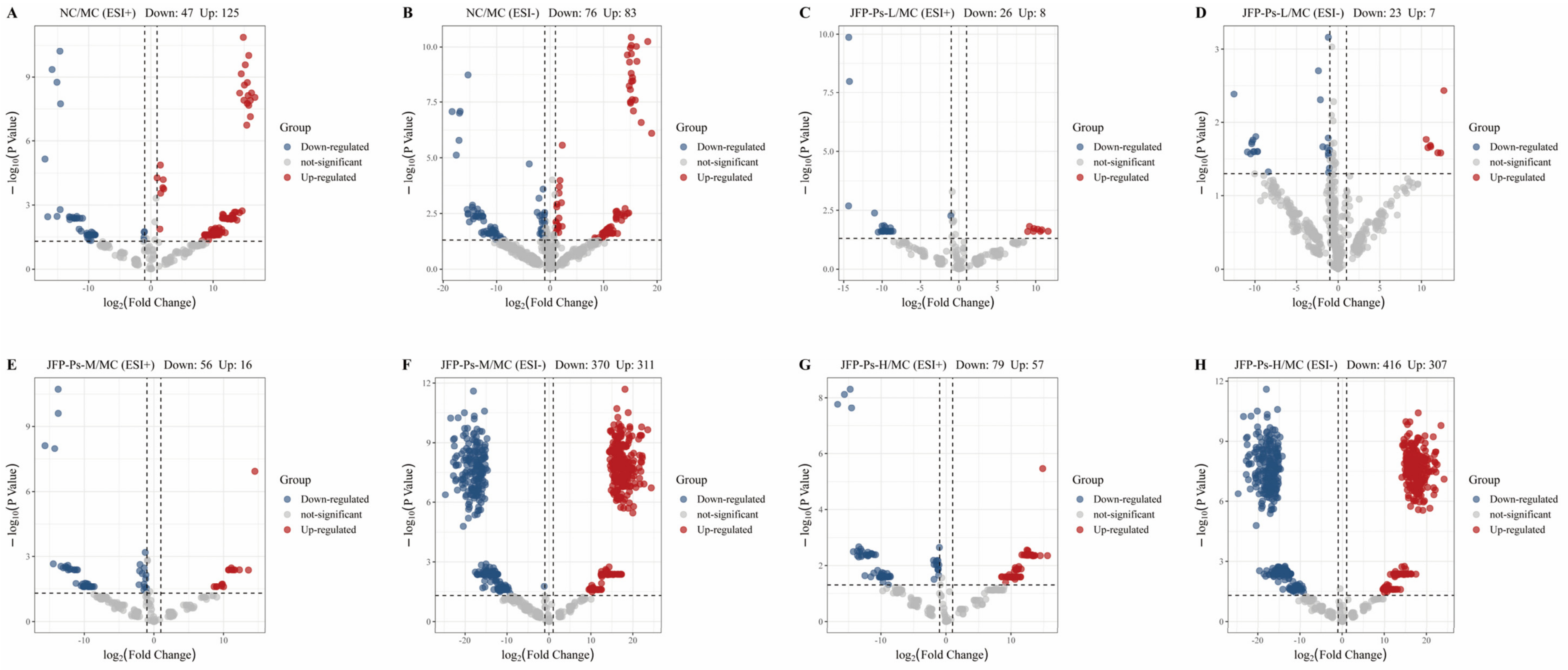

Figure S5 Volcano plot of differential metabolites. (A), (C), (E) and (G) are positive ion modes. (B), (D), (F), and (H) are the negative ion mode

Table S1 Fecal differential metabolites in NC, MC, and JFP-Ps-H groups

No.	Compound	Mode	Formula	HMDBID	RT (min)	Mass	NC/MC		JFP-Ps-H/MC	
							Log FC	Change	Log FC	Change
1	Valyl-Valine	ESI+	$C_{10}H_{20}N_2O_3$	HMDB29140	7.39	216.1467	14.527844	up	0	—
2	Tryptophyl-Isoleucine	ESI+	$C_{17}H_{23}N_3O_3$	HMDB29086	8.98	317.1752	15.034807	up	0	—
3	Ganglioside GD1b (d18:1/12:0)	ESI+	$C_{79}H_{137}N_3O_{39}$	HMDB11822	9.84	1751.8899	16.17651975	up	0	—
4	Phenylalanyl-Alanine	ESI+	$C_{12}H_{16}N_2O_3$	HMDB28988	6.11	236.1169	1.5261649	up	0.729581679	up
5	Valyl-Glutamine	ESI+	$C_{10}H_{19}N_3O_4$	HMDB29125	5.05	245.1385	14.6744	up	13.16961417	up
6	Pseudoecgonine	ESI+	$C_9H_{15}NO_3$	HMDB06348	5.39	185.1058	0	—	7.3584094	up
7	Isoleucyl-Leucine	ESI+	$C_{12}H_{24}N_2O_3$	HMDB28911	6.73	244.1794	13.18510392	up	7.214352133	up
8	Aklavinone	ESI+	$C_{22}H_{20}O_8$		10.66	412.1145	11.551977	up	12.2317505	up
9	6-Deoxyfagomine	ESI+	$C_6H_{13}NO_2$	HMDB36382	5.25	131.095	13.17129042	up	2.49551155	up
10	Indoleacrylic acid	ESI+	$C_{11}H_9NO_2$	HMDB00734	6.10	165.0791	11.775587	up	10.014887	up
11	3,5-Didecanoylpyridine	ESI+	$C_{25}H_{41}NO_2$	HMDB35515	20.17	387.3132	11.92407658	up	4.562925333	up
12	N-Acetyl-leu-leu-tyr-amide	ESI+	$C_{23}H_{36}N_4O_5$		9.60	448.2695	13.40240167	up	0	—
13	Methionyl-Isoleucine	ESI+	$C_{11}H_{22}N_2O_3S$	HMDB28976	6.02	262.1355	12.63594475	up	0	—
14	LysoPE(18:2(9Z,12Z)/0:0)	ESI+	$C_{23}H_{44}NO_7P$	HMDB11507	18.29	477.2857	9.7532712	up	0	—
15	3-Hydroxynonyl acetate	ESI+	$C_{11}H_{22}O_3$	HMDB32443	1.37	224.1407	8.6591518	up	6.50068585	up
16	Alanyl-Lysine	ESI+	$C_9H_{19}N_3O_3$	HMDB28692	1.36	217.1433	9.273635367	up	2.3097583	up
17	(9Z,11R,12S,13S,15Z)-12,13-Epoxy-11-hydroxy-9,15-octadecadienoic acid	ESI+	$C_{18}H_{30}O_4$	HMDB33505	19.95	310.2158	11.40926392	up	5.579503833	up
18	L-Pyroglutamic acid	ESI+	$C_5H_7NO_3$	HMDB0000805	2.24	129.0433	2.296594	up	9.702455	up
19	Tryptophan	ESI+	$C_{11}H_{12}N_2O_2$	HMDB0000929	6.10	226.0733	-4.7164493	down	-11.394096	down

Continued table S1 Fecal differential metabolites in NC, MC, and JFP-Ps-H groups

NO.	Compound	Mode	Formula	HMDBID	RT (min)	Mass	NC/MC		JFP-Ps-H/MC	
							Log FC	Change	Log FC	Change
20	5-(2-Hydroxyethyl)-4-methylthiazole	ESI+	C_6H_9NOS	HMDB0032985	3.96	143.0405	0.039390326	up	12.572438	up
21	Puromycin	ESI+	$C_{22}H_{29}N_7O_5$	HMDB0256926	1.79	493.2021	-9.555646	down	-11.969058	down
22	9-chloro-10-hydroxy-hexadecanoic acid	ESI+	$C_{16}H_{31}ClO_3$	LMFA01090044	12.55	306.1952	-2.4795153	down	10.98033787	up
23	OryzalideB	ESI+	$C_{19}H_{28}O_4$	HMDB37592	5.91	342.1807	-13.047373	down	-13.047373	down
24	Prolyl-Alanine	ESI+	$C_8H_{14}N_2O_3$	HMDB29010	7.06	186.1008	-7.7572937	down	-9.788935	down
25	Nicotinuric acid	ESI+	$C_8H_8N_2O_3$	HMDB03269	6.59	180.0542	2.3043916	up	9.733729	up
26	2'-Deoxyadenosine		$C_{11}H_{15}N_5O_3$		4.50	265.1179	2.422829217	up	10.37502703	up
27	Indole	ESI+	CH_7N	HMDB00738	6.11	117.059	-0.1254162	down	-7.231327	down
28	Xanthurenic acid	ESI+	$C_{10}H_7NO_4$	HMDB00881	5.88	205.0379	0	—	10.284391	up
29	Taurallocholic acid	ESI+	$C_{26}H_{45}NO_7S$	HMDB00922	10.92	515.2924	-8.1000973	down	-5.4984136	down
30	Uric acid	ESI-	$C_5H_4N_4O_3$	HMDB00289	1.74	168.0286	-4.5675022	down	-0.779365	down
31	Palmitic Acid	ESI-	$C_{16}H_{32}O_2$	HMDB00220	26.67	256.2409	0.8884597	up	-14.600051	down
32	N-Oleoyl Dopamine	ESI-	$C_{26}H_{43}NO_3$		25.18	417.3254	-13.995832	down	-2.7598565	down
33	Glutamyl-arginine	ESI-	$C_{11}H_{21}N_5O_5$	HMDB28813	9.73	349.1579	12.30678492	up	4.836052233	up
34	2-Dehydro-3-deoxy-D-galactonate	ESI-	$C_6H_{10}O_6$	HMDB0001353	1.74	178.0484	-14.358494	down	-3.1245706	down
35	Beta-Cortol	ESI-	$C_{21}H_{36}O_5$	HMDB05821	22.72	368.2584	-1.4490822	down	-18.063078	down
36	Psychosine	ESI-	$C_{24}H_{47}NO_7$	HMDB00648	22.71	507.3401	-0.9082928	down	-17.719336	down
37	Leukotriene E3	ESI-	$C_{23}H_{39}NO_5S$	HMDB02355	9.60	501.2765	-11.094137	down	-13.6227	down

Continued table S1 Fecal differential metabolites in NC, MC, and JFP-Ps-H groups

No.	Compound	Mode	Formula	HMDBID	RT (min)	Mass	NC/MC		JFP-Ps-H/MC	
							Log FC	Change	Log FC	Change
38	9,10,13-Trihydroxystearic acid	ESI-	$C_{18}H_{36}O_5$	HMDB30935	13.05	332.2548	13.944369	up	15.911865	up
39	1-Naphthylacetylspermine	ESI-	$C_{22}H_{34}N_4O$		18.80	416.2755	11.186954	up	15.332336	up
40	Dihomo-γ-Linolenic Acid	ESI-	$C_{20}H_{34}O_2$	LMFA01030158	26.64	306.2562	-9.3102921	down	-5.9717058	down
41	Isoursodeoxycholic acid	ESI-	$C_{24}H_{40}O_4$	HMDB00686	16.92	392.2914	10.4891621	up	2.581002	up
42	Tetrahydrocortisol	ESI-	$C_{21}H_{34}O_5$	HMDB00949	20.78	366.2407	8.991491767	up	2.605619167	up
43	11(Z),14(Z)-Eicosadienoic Acid	ESI-	$C_{20}H_{36}O_2$	HMDB05060	27.96	308.2727	-8.36534056	down	-13.343213	down
44	LysoPE(18:3(6Z,9Z,12Z)/0:0)	ESI-	$C_{23}H_{42}NO_7P$	HMDB11508	19.50	521.269	3.034324267	up	17.67568218	up
45	1,2-Diacyl-glycerophospholipid	ESI-	$C_{31}H_{61}O_8P$	HMDB02079	15.05	652.4298	-6.19550365	down	-17.0025794	down
46	MG(18:2(9Z,12Z)/0:0/0:0)	ESI-	$C_{21}H_{38}O_4$	HMDB11568	24.07	400.2809	0	—	19.6777615	up
47	Enterolactone	ESI-	$C_{18}H_{18}O_4$	HMDB06101	13.43	298.1192	0	—	18.03164283	up
48	Taurochenodeoxycholic acid	ESI-	$C_{26}H_{45}NO_6S$	LMST05040005	14.56	499.2933	0	—	17.05169797	up
49	PG(18:3(9Z,12Z,15Z)/20:4(5Z,8Z,11Z,14Z))	ESI-	$C_{44}H_{73}O_{10}P$	HMDB10685	9.60	792.4972	5.029196933	up	15.26316702	up
50	Nutriacholic acid	ESI-	$C_{24}H_{38}O_4$	HMDB00467	17.25	436.2808	0	—	17.30354928	up
51	LysoPE(20:2(11Z,14Z)/0:0) -18.758165	ESI-	$C_{25}H_{48}NO_7P$	HMDB11513	18.36	565.3359	0	—	13.51739967	up
52	Taurallocholic acid	ESI-	$C_{26}H_{45}NO_7S$	HMDB00922	10.62	515.2893	8.900185928	up	11.98958327	up
53	Prednisolone tebutate	ESI-	$C_{27}H_{38}O_6$		18.91	458.2716	-2.5102658	down	-15.907505	down
54	Ursodeoxycholic Acid	ESI-	$C_{24}H_{40}O_4$	HMDB00664	16.73	392.2904	0	—	18.94731508	up
55	12-ketolithocholic acid	ESI-	$C_{24}H_{38}O_5$	HMDB00400	15.41	406.2704	5.271321667	up	17.520813	up

Continued table S1 Fecal differential metabolites in NC, MC, and JFP-Ps-H groups

No.	Compound	Mode	Formula	HMDBID	RT (min)	Mass	NC/MC		JFP-Ps-H/MC	
							LogFC	Change	LogFC	Change
56	LysoPE(0:0/15:0)	ESI-	$C_{20}H_{42}NO_7P$	HMDB11472	18.27	439.2683	0	—	21.68884517	up
57	Ganglioside GA2 (d18:1/18:0)	ESI-	$C_{56}H_{104}N_2O_{18}$	HMDB04891	22.32	1092.7253	0	—	17.31498517	up
58	PIP2(16:1(9Z)/16:0)	ESI-	$C_{41}H_{79}O_{19}P_3$	HMDB10051	13.01	1014.4446	-2.2103284	down	-17.0837998	down
59	3-Hydroxyanthranilate	ESI-	$C_7 H_7 N O_4$		1.51	169.0371	16.26881733	up	15.143276	up
60	Chenodeoxycholic acid	ESI-	$C_{24} H_{40} O_4$	HMDB00518	24.16	424.2804	10.69947727	up	16.65015783	up
61	3a,7a-Dihydroxycoprostanic acid	ESI-	$C_{27} H_{46} O_4$	HMDB0000359	23.69	434.3379	0	—	16.8191822	up
